# Brain Computer Interfaces, a Review

**DOI:** 10.3390/s120201211

**Published:** 2012-01-31

**Authors:** Luis Fernando Nicolas-Alonso, Jaime Gomez-Gil

**Affiliations:** Department of Signal Theory, Communications and Telematics Engineering, University of Valladolid, Valladolid 47011, Spain; E-Mail: jgomgil@tel.uva.es

**Keywords:** brain-computer interface (BCI), electroencephalography (EEG), rehabilitation, artifact, neuroimaging, brain-machine interface, collaborative sensor system

## Abstract

A brain-computer interface (BCI) is a hardware and software communications system that permits cerebral activity alone to control computers or external devices. The immediate goal of BCI research is to provide communications capabilities to severely disabled people who are totally paralyzed or ‘locked in’ by neurological neuromuscular disorders, such as amyotrophic lateral sclerosis, brain stem stroke, or spinal cord injury. Here, we review the state-of-the-art of BCIs, looking at the different steps that form a standard BCI: signal acquisition, preprocessing or signal enhancement, feature extraction, classification and the control interface. We discuss their advantages, drawbacks, and latest advances, and we survey the numerous technologies reported in the scientific literature to design each step of a BCI. First, the review examines the neuroimaging modalities used in the signal acquisition step, each of which monitors a different functional brain activity such as electrical, magnetic or metabolic activity. Second, the review discusses different electrophysiological control signals that determine user intentions, which can be detected in brain activity. Third, the review includes some techniques used in the signal enhancement step to deal with the artifacts in the control signals and improve the performance. Fourth, the review studies some mathematic algorithms used in the feature extraction and classification steps which translate the information in the control signals into commands that operate a computer or other device. Finally, the review provides an overview of various BCI applications that control a range of devices.

## 1. Introduction

A brain computer interface (BCI), also referred to as a brain machine interface (BMI), is a hardware and software communications system that enables humans to interact with their surroundings, without the involvement of peripheral nerves and muscles, by using control signals generated from electroencephalographic activity. BCI creates a new non-muscular channel for relaying a person’s intentions to external devices such as computers, speech synthesizers, assistive appliances, and neural prostheses. That is particularly attractive for individuals with severe motor disabilities. Such an interface would improve their quality of life and would, at the same time, reduce the cost of intensive care.

A BCI is an artificial intelligence system that can recognize a certain set of patterns in brain signals following five consecutive stages: signal acquisition, preprocessing or signal enhancement, feature extraction, classification, and the control interface [1]. The signal acquisition stage captures the brain signals and may also perform noise reduction and artifact processing. The preprocessing stage prepares the signals in a suitable form for further processing. The feature extraction stage identifies discriminative information in the brain signals that have been recorded. Once measured, the signal is mapped onto a vector containing effective and discriminant features from the observed signals. The extraction of this interesting information is a very challenging task. Brain signals are mixed with other signals coming from a finite set of brain activities that overlap in both time and space. Moreover, the signal is not usually stationary and may also be distorted by artifacts such as electromyography (EMG) or electrooculography (EOG). The feature vector must also be of a low dimension, in order to reduce feature extraction stage complexity, but without relevant information loss. The classification stage classifies the signals taking the feature vectors into account. The choice of good discriminative features is therefore essential to achieve effective pattern recognition, in order to decipher the user’s intentions. Finally the control interface stage translates the classified signals into meaningful commands for any connected device, such as a wheelchair or a computer.

BCI technology has traditionally been unattractive for serious scientific investigation. The idea of successfully deciphering thoughts or intentions by means of brain activity has often been rejected in the past as very strange and remote. Hence investigation in the field of brain activity has usually been limited to the analysis of neurological disorders in the clinic or to the exploration of brain functions in the laboratory. The BCI design was considered too complex, because of the limited resolution and reliability of information that was detectable in the brain and its high variability. Furthermore, BCI systems require real-time signal processing, and up until recently the requisite technology either did not exist or was extremely expensive [2].

However, this context has undergone radical change over the last two decades. BCI research, which was confined to only three groups 20 years ago and only six to eight groups 10 years ago, is now a flourishing field with more than 100 active research groups all over the World studying the topic [3]. The number of articles published regarding neural interface technology has increased exponentially over the past decade [4]. Successful studies on brain signal phenomena have lent further weight to these advances. The development of more and more inexpensive computer hardware and software has allowed more sophisticated online analysis. Likewise, the chances of using BCIs as auxiliary technology that might serve severely disabled people has increased social acceptance in this field and the need to accelerate its progress. Interest in this technology is now found outside of the laboratory or the clinic. Small specialized companies such as Emotiv [5] or Neurosky [6] have already developed some initial applications oriented towards the general public. Nevertheless, despite these advances, most BCI-based applications are still limited to the laboratory. Broader applicability of BCIs requires greater ease of use, which in turn means reducing time spent on preparation, training and calibration [7].

BCI research is a relatively young multidisciplinary field integrating researchers from neuroscience, physiology, psychology, engineering, computer science, rehabilitation, and other technical and health-care disciplines. As a result, in spite of some notable advances, a common language has yet to emerge, and existing BCI technologies vary, which makes their comparison difficult and, in consequence, slows down the research. The community of BCI researchers has therefore stressed the need to establish a general framework for BCI design [8]. Mason *et al.* [9], for example, proposed a new functional model for BCI systems and taxonomy design.

This review of the state-of-the-art of BCI systems is arranged as follows: Section 2 discusses existing neuroimaging approaches to BCIs and Section 3 describes the most commonly found control signals in BCI systems. Section 4 briefly explains certain types of BCIs. Sections 5, 6 and 7, respectively, cover the different signal processing methods used for feature extraction, artifact reduction and feature classification. Section 8 provides an overview of BCI applications and, finally, the conclusions are drawn in Section 9.

## 2. Neuroimaging Approaches in BCIs

BCIs use brain signals to gather information on user intentions. To that effect, BCIs rely on a recording stage that measures brain activity and translates the information into tractable electrical signals. Two types of brain activities may be monitored: (i) electrophysiological and (ii) hemodynamic.

Electrophysiological activity is generated by electro-chemical transmitters exchanging information between the neurons. The neurons generate ionic currents which flow within and across neuronal assemblies. The large variety of current pathways can be simplified as a dipole conducting current from a source to a sink through the dendritic trunk. These intracellular currents are known as primary currents. Conservation of electric charges means that the primary currents are enclosed by extracellular current flows, which are known as secondary currents [10]. Electrophysiological activity is measured by electroencephalography, electrocorticography, magnetoencephalography, and electrical signal acquisition in single neurons.

The hemodynamic response is a process in which the blood releases glucose to active neurons at a greater rate than in the area of inactive neurons. The glucose and oxygen delivered through the blood stream results in a surplus of oxyhemoglobin in the veins of the active area, and in a distinguishable change of the local ratio of oxyhemoglobin to deoxyhemoglobin [11]. These changes can be quantified by neuroimaging methods such as functional magnetic resonance and near infrared spectroscopy. These kinds of methods are categorized as indirect, because they measure the hemodynamic response, which, in contrast to electrophysiological activity, is not directly related to neuronal activity.

Most current BCIs obtain the relevant information from the brain activity through electroencephalography. Electroencephalography is by far the most widely used neuroimaging modality, owing to its high temporal resolution, relative low cost, high portability, and few risks to the users. BCIs based on electroencephalography consist of a set of sensors that acquire electroencephalography signals from different brain areas. However, the quality of electroencephalography signals is affected by scalp, skull, and many other layers as well as background noise. Noise is key to electroencephalography and to other neuroimaging methods, insofar as it reduces the SNR and therefore the ability to extract meaningful information from the recorded signals.

Non-invasive approaches have successfully been used by severely and partially paralyzed patients to reacquire basic forms of communication and to control neuroprostheses and wheelchairs [12–14]. Despite the outstanding utility of non-invasive approaches in BCI applications, motor recovery has been limited, because of the need for brain signals with a higher resolution. Invasive recording methods such as electrocorticography or intracortical neuron recording were introduced, in an effort to improve the quality of brain signals monitored by BCIs. Most researchers agree that movement restoration through prostheses with multiples degrees of freedom can only be achieved through invasive approaches [15]. It is unlikely that the power of non-invasive modalities will be enhanced in the near future. Accordingly, it would appear that invasive modalities are indispensable for accurate neuroprostheses control. Nevertheless, this issue is not yet entirely clear and some opinions disagree with this conjecture. Contrary to established opinion, Wolpaw [3] suggested that performance in multidimensional control may be independent of the recording method. Further refinements of recording and analysis techniques will probably increase the performance of both invasive and non-invasive modalities. However, the latest studies in neuroprostheses control appear to indicate that invasive modalities have inherent advantages in neuroprosthesis control applications [4].

Invasive modalities need to implant microelectrode arrays inside the skull that involves significant health risks, which restricts their use to experimental settings. Two invasive modalities can be found in BCI research: electrocorticography, which places electrodes on the surface of the cortex, either outside the dura mater (epidural electrocorticography) or under the dura mater (subdural electrocorticography), and intracortical neuron recording which implants electrodes inside the cortex. Several issues had to be addressed, before they become suitable for long-term applications. First, tissue acceptance of the microelectrode has to be addressed, for which reason proposals exist for electrodes with neurotropic mediums that promote neuronal growth to improve biocompatibility [16]. Perhaps, the future of nanotechnologies that might develop nano-detectors to be implanted inertly in the brain, may provide a definite solution to the problems of long-term invasive applications. Second, a link between the microelectrode and external hardware that uses wireless technology is needed to reduce the risks of infection. Wireless transmission of neuronal signals has already been tested in animals [17]. And third, continuous stress caused by plugging and unplugging the recording system may lead to tissue damage or system failure.

Each neuroimaging modality is explained below. Firstly, electrophysiological methods such as electroencephalography, electrocorticography, magnetoencephalography, and electrical signal acquisition in single neurons will be discussed. Secondly, metabolic methods such as functional magnetic resonance and near infrared spectroscopy will be described. Finally, functional imaging modalities are listed in Table 1, along with information related to activity measured, temporal and spatial resolutions, safety, and portability.

### 2.1. Electroencephalography (EEG)

EEG measures electric brain activity caused by the flow of electric currents during synaptic excitations of the dendrites in the neurons and is extremely sensitive to the effects of secondary currents [10]. EEG signals are easily recorded in a non-invasive manner through electrodes placed on the scalp, for which that reason it is by far the most widespread recording modality. However, it provides very poor quality signals as the signals have to cross the scalp, skull, and many other layers. This means that EEG signals in the electrodes are weak, hard to acquire and of poor quality. This technique is moreover severely affected by background noise generated either inside the brain or externally over the scalp.

The EEG recording system consists of electrodes, amplifiers, A/D converter, and a recording device. The electrodes acquire the signal from the scalp, the amplifiers process the analog signal to enlarge the amplitude of the EEG signals so that the A/D converter can digitalize the signal in a more accurate way. Finally, the recording device, which may be a personal computer or similar, stores, and displays the data.

The EEG signal is measured as the potential difference over time between signal or active electrode and reference electrode. An extra third electrode, known as the ground electrode, is used to measure the differential voltage between the active and the reference points. The minimal configuration for EEG measurement therefore consists of one active, one reference, and one ground electrode. Multi-channel configurations can comprise up to 128 or 256 active electrodes [18]. These electrodes are usually made of silver chloride (AgCl) [19]. Electrode-scalp contact impedance should be between 1 kΩ and 10 kΩ to record an accurate signal [20]. The electrode-tissue interface is not only resistive but also capacitive and it therefore behaves as a low pass filter. The impedance depends on several factors such as the interface layer, electrode surface area, and temperature [20]. EEG gel creates a conductive path between the skin and each electrode that reduces the impedance. Use of the gel is cumbersome, however, as continued maintenance is required to assure a relatively good quality signal. Electrodes that do not need to use of gels, called ‘dry’ electrodes, have been made with other materials such as titanium and stainless-steel [21]. These kinds of electrodes may be ‘dry’ active electrodes, which have preamplification circuits for dealing with very high electrode/skin interfacial impedances [21,22], or ‘dry’ passive electrodes, which have no active circuits, but are linked to EEG recording systems with ultra-high input impedance [23].

The amplitude of electrical bio-signals is in the order of microvolts. Consequently, the signal is very sensitive to electronic noise. External sources such power-lines may generate background noise and thermal, shot, flicker, and burst noises are generated by internal sources [24]. Design considerations should be addressed to reduce the effects of the noise, such as electromagnetic interference shielding or reduction for common mode signal, amongst others [20].

EEG comprises a set of signals which may be classified according to their frequency. Well-known frequency ranges have been defined according to distribution over the scalp or biological significance. These frequency bands are referred to as delta (δ), theta (θ), alpha (α), beta (β), and gamma (γ) from low to high, respectively. Relevant characteristics of these bands are detailed below.

The delta band lies below 4 Hz, and the amplitude of delta signals detected in babies decreases as they age. Delta rhythms are usually only observed in adults in deep sleep state and are unusual in adults in an awake state. A large amount of delta activity in awake adults is abnormal and is related to neurological diseases [25]. Due to low frequency, it is easy to confuse delta waves with artifact signals, which are caused by the large muscles of the neck or jaw.

Theta waves lie within the 4 to 7 Hz range. In a normal awake adult, only a small amount of theta frequencies can be recorded. A larger amount of theta frequencies can be seen in young children, older children, and adults in drowsy, meditative or sleep states [25]. Like delta waves, a large amount of theta activity in awake adults is related to neurological disease [25]. Theta band has been associated with meditative concentration [26,27] and a wide range of cognitive processes such as mental calculation [28], maze task demands [29], or conscious awareness [30].

Alpha rhythms are found over the occipital region in the brain [31]. These waves lie within the 8 to 12 Hz range. Their amplitude increases when the eyes close and the body relaxes and they attenuate when the eyes open and mental effort is made [32]. These rhythms primarily reflect visual processing in the occipital brain region and may also be related to the memory brain function [33]. There is also evidence that alpha activity may be associated with mental effort. Increasing mental effort causes a suppression of alpha activity, particularly from the frontal areas [34]. Consequently, these rhythms might be useful signals to measure mental effort. Mu rhythms may be found in the same range as alpha rhythms, although there are important physiological differences between both. In contrast to alpha rhythms, mu rhythms are strongly connected to motor activities and, in some cases, appear to correlate with beta rhythms [31,35].

Beta rhythms, within the 12 to 30 Hz range, are recorded in the frontal and central regions of the brain and are associated with motor activities. Beta rhythms are desynchronized during real movement or motor imagery [36]. Beta waves are characterized by their symmetrical distribution when there is no motor activity. However, in case of active movement, the beta waves attenuate, and their symmetrical distribution changes [36].

Gamma rhythms belong to the frequency range from 30 to 100 Hz. The presence of gamma waves in the brain activity of a healthy adult is related to certain motor functions or perceptions, among others [37]. Some experiments have revealed a relationship in normal humans between motor activities and gamma waves during maximal muscle contraction [38]. This gamma band coherence is replaced by a beta band coherence during weak contractions, suggesting a correlation between gamma or beta cortical oscillatory activity and force [39]. Also, several studies have provided evidence for the role of gamma activity in the perception of both visual and auditory stimuli [37,40–42]. Gamma rhythms are less commonly used in EEG-based BCI systems, because artifacts such as electromyography (EMG) or electrooculography (EOG) are likely to affect them [43]. Nevertheless, this range is attracting growing attention in BCI research because, compared to traditional beta and alpha signals, gamma activity may increase the information transfer rate and offer higher spatial specifity [44,45].

**Figure 1.**
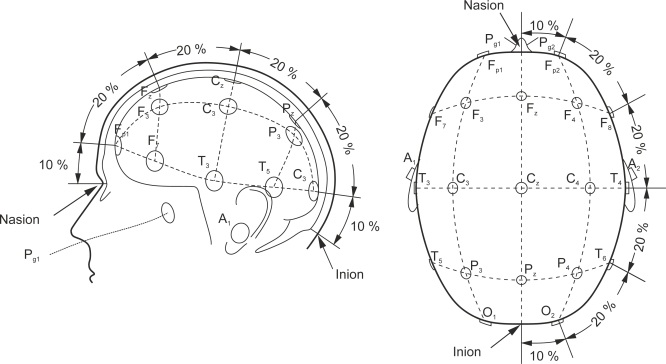
Electrode placement over scalp.

As explained above, EEG is recorded by electrodes. The electrodes placed over the scalp are commonly based on the International 10–20 system [46], which has been standardized by the American Electroencephalographic Society. The 10–20 system uses two reference points in the head to define the electrode location. One of these reference points is the nasion, located at the top of the nose at the same level as the eyes. The other reference point is the inion, which is found in the bony lump at the base of the skull. The transverse and median planes divide the skull from these two points. The electrode locations are determined by marking these planes at intervals of 10% and 20% (Figure 1). The letters in each location corresponds to specific brain regions in such a way that A represents the ear lobe, C the central region, P_g_ the nasopharyngeal, P the parietal, F the frontal, F_p_ the frontal polar, and O the occipital area.

### 2.2. Magnetoencephalography (MEG)

MEG is a non-invasive imaging technique that registers the brain’s magnetic activity by means of magnetic induction. MEG measures the intracellular currents flowing through dendrites which produce magnetic fields that are measurable outside of the head [47]. The neurophysiological processes that produce MEG signals are identical to those that produce EEG signals. Nevertheless, while EEG is extremely sensitive to secondary current sources, MEG is more sensitive to those of primary currents [10]. The advantage of MEG is that magnetic fields are less distorted by the skull and scalp than electric fields [48].

Magnetic fields are detected by superconducting quantum interferences devices, which are extremely sensitive to magnetic disturbances produced by neural activity [49]. The electronic equipment that measures magnetic brain activity is cooled to almost −273 degrees Celsius to facilitate sensor superconductivity. MEG requires effective shielding from electromagnetic interferences. The electronic equipment is installed inside a magnetically shielded room, which attenuates the effects of magnetic fields from external sources.

MEG provides signals with higher spatiotemporal resolution than EEG, which reduces the training time needed to control a BCI and speeds up reliable communications [50]. MEG has also been successfully used to localize active regions inside the brain [51]. In spite of these advantageous features, MEG is not often used in BCI design because MEG technology is too bulky and expensive to become an acquisition modality suitable for everyday use. In 2005, Lal *et al.* [52] presented the first online MEG-based BCI. Although further studies have followed [53–57], MEG-based BCIs, as compared to EEG-based BCIs, are still at an early stage.

### 2.3. Electrocorticography (ECoG)

ECoG is a technique that measures electrical activity in the cerebral cortex by means of electrodes placed directly on the surface of the brain. Compared to EEG, ECoG provides higher temporal and spatial resolution as well as higher amplitudes and a lower vulnerability to artifacts such as blinks and eye movement [58]. However, ECoG is an invasive recording modality which requires a craniotomy to implant an electrode grid, entailing significant health hazards. For that reason, the first studies on ECoG were with animals. Early studies involving animals evaluated the long-term stability of the signals from the brain that ECoG could acquire [59–62]. The results showed that subdural electrodes could provide stable signals over several months. Nevertheless, the long-term stability of the signals acquired by ECoG is currently unclear. More recent experiments with monkeys have shown that ECoG can perform at a high level for months without any drift in accuracy or recalibration [63]. The hand positions and arm joint angles could be successfully decoded during asynchronous movements. These studies have also developed minimally invasive protocols to implant the ECoG probes [64].

In humans, ECoG has been used for the analysis of alpha and beta waves [65] or gamma waves [66,67] produced during voluntary motor action. With regard to the use of ECoG in BCIs systems, Levine *et al.* [68] designed a BCI which classified motor actions on the basis of the identification of the event-related potentials (ERP) using ECoG. Leuthardt *et al.* [69] showed for the first time that an ECoG-based BCI could provide information to control a one-dimensional cursor, as this information is more precise and more quickly acquired than by EEG-based BCIs. Some years later, Schalk *et al.* [70] presented a more advanced ECoG-based BCI which allowed the user to control a two-dimensional cursor. The results of all these studies might make it more feasible for people with severe motor disabilities to use ECoG-based BCIs for their communication and control needs.

### 2.4. Intracortical Neuron Recording

Intracortical neuron recording is a neuroimaging technique that measures electrical activity inside the gray matter of the brain. It is an invasive recording modality that needs to implant microelectrode arrays inside the cortex to capture spike signals and local field potentials from neurons.

Three signals can be obtained by intracortical neuron recording: single-unit activity (SUA), multi-unit activity (MUA), and local field potentials (LFPs) [47]. SUA is obtained by high-pass filtering (>300 Hz) of the signal of a single neuron. MUA is obtained in the same way, but the signals may come from multiple neurons. LFPs are extracted by low-pass filtering (<300 Hz) of the neuron activity in the vicinity of an electrode tip. LFPs are analog signals whereas SUA and MUA measure the spiking activity of single neurons and can be reduced to discrete events in time [47].

Intracortical neuron recording provides much higher spatial and temporal resolution than EEG recording. Hence the intracortical signals may be easier to use than EEG signals. However, signal quality may be affected by the reaction of cerebral tissue to the implanted recording microelectrode [71] and by changes in the sensitivity of the microelectrode, which may be progressively damaged over the course of days and years [72]. The user can naturally adapt to these slow changes in the relative sensitivity of the microelectrode, without the need for specific retraining. Nevertheless, periodic recalibrations of electrode sensitivity may be necessary [73].

The first attempts in the intracortical neuron recording field were made in animals. Multielectrode arrays have been used to record neural activity from the motor cortex in monkeys or rats during learned movements [74–76]. These initial studies have shown that intracortical neuron recordings can indicate the nature of a movement and its direction. These studies do not reveal whether the same patterns will be present when the real movements are not made. In that regard, Taylor and Schwartz [77] experimented with rhesus macaques, which made real and virtual arm movements in a computer. The results suggested that the same patterns persisted. The most recent studies with monkeys investigated the control of prosthetic devices for direct real-time interaction with the physical environment [78–81].

With regard to the application of intracortical neuron recording in BCI systems, microelectrode arrays such as the Utah Intracortical Electrode Array (UIEA) have been reported as a suitable means of providing simultaneous and proportional control of a large number of external devices [72]. Also, Kennedy *et al.* [82] employed cortical control signals to design a BCI that allowed users to control cursor movement and flexion of a cyber-digit finger on a virtual hand.

### 2.5. Functional Magnetic Resonance Imaging (fMRI)

fMRI is a non-invasive neuroimaging technique which detects changes in local cerebral blood volume, cerebral blood flow and oxygenation levels during neural activation by means of electromagnetic fields. fMRI is generally performed using MRI scanners which apply electromagnetic fields of strength in the order of 3T or 7T. The main advantage of the use of fMRI is high space resolution. For that reason, fMRI have been applied for localizing active regions inside the brain [83]. However, fMRI has a low temporal resolution of about 1 or 2 seconds. Additionally, the hemodynamic response introduces a physiological delay from 3 to 6 seconds [84]. fMRI appears unsuitable for rapid communication in BCI systems and is highly susceptible to head motion artifacts.

In BCI systems, fMRI is typically used to measure the Blood Oxygen Level Dependent (BOLD) during neuronal activation [85]. Although the BOLD signal is not directly related to neuronal activity, a correspondence between both does exist [86]. The use of fMRI in BCI technology is relatively recent. Before the emergence of real-time fMRI, brain activity recording by fMRI has traditionally taken a long time. The data acquired by fMRI techniques were processed offline and the results only became available after several hours or even days [87]. fMRI-based BCIs have been made possible, thanks to the development of real-time fMRI [84,88,89]. The information transfer rate in fMRI-based BCIs is between 0.60 and 1.20 bits/min [90]. Non-clinical fMRI applications are not expected because fMRI requires overly bulky and expensive hardware.

### 2.6. Near Infrared Spectroscopy (NIRS)

NIRS is an optical spectroscopy method that employs infrared light to characterize noninvasively acquired fluctuations in cerebral metabolism during neural activity. Infrared light penetrates the skull to a depth of approximately 1–3 cm below its surface, where the intensity of the attenuated light allows alterations in oxyhemoglobin and deoxyhemoglobin concentrations to be measured. Due to shallow light penetration in the brain, this optical neuroimaging technique is limited to the outer cortical layer. In a similar way to fMRI, one of the major limitations of NIRS is the nature of the hemodynamic response, because vascular changes occur a certain number of seconds after its associated neural activity [91]. The spatial resolution of NIRS is quite low, in the order of 1 cm [92]. Nevertheless, NIRS offers low cost, high portability, and an acceptable temporal resolution in the order of 100 milliseconds [93].

A NIRS system consists of a light source, a driving electronic device, a light detector, signal processing devices, and a recording device. The light source is an infrared emitting diode (IRED) placed in direct contact with the scalp. The driving electronic device is an electronic circuit that controls the IRED in order to modulate the light. The light detector is a photodiode placed right next to the light source. The signal processing devices are amplifiers and filters that process the electrical signal and reduce the noise due to ambient light. The recording device is a personal computer or any other device that digitalizes, stores, and displays the electrical signal.

Ensuring good coupling light from the optical sources and detectors to and from the subject’s head is not a trivial issue. Head motions or hair obstruction can worsen performance and signal quality [91]. Good quality signals and noise reduction, especially background noise induced by head motions, are important requirements in real time BCI systems. Hair obstruction can be overcome by combing the hair out of the photons’ path by means of hair gel and hair clips [91]. Noise can be reduced partially by bandpass filtering, moving averaging, and Wiener filtering. These classes of algorithms usually fail to remove abrupt spike-like noise produced by head motion [94]. Head motion artifacts can be minimized by ensuring rigid optode positioning. Solutions have been introduced that are based on helmets, thermoplastic molded to the contours of each subject’s head, spring-loaded fibers attached to semi-rigid plastic forms, and fibers embedded in neoprene rubber forms [95]. Background noise effects can also be attenuated by exploiting the strong statistical association between oxygenated and deoxygenated hemoglobin dynamics [96].

Although NIRS is relatively new measurement modality, NIRS promises to be a potent neuroimaging modality for future applicability to BCIs [91,97]. NIRS provides now a low information transfer rate of about 4 bits/min but it would be increased in the future [98]. This neuroimaging modality might be a good alternative to EEG, as neither conductive gel nor corrosive electrodes are required. Nevertheless, communication speeds in NIRS-based BCIs are limited due to the inherent delays of the hemodynamic response. Some studies have already demonstrated the feasibility of mental task detection through NIRS-derived optical responses [93,99,100].

## 3. Control Signal Types in BCIs

The purpose of a BCI is to interpret user intentions by means of monitoring cerebral activity. Brain signals involve numerous simultaneous phenomena related to cognitive tasks. Most of them are still incomprehensible and their origins are unknown. However, the physiological phenomena of some brain signals have been decoded in such way that people may learn to modulate them at will, to enable the BCI systems to interpret their intentions. These signals are regarded as possible control signals in BCIs.

Numerous studies have described a vast group of brain signals that might serve as control signals in BCI systems. Nevertheless, only those control signals employed in current BCI systems will be discussed below: visual evoked potentials, slow cortical potentials, P300 evoked potentials, and sensorimotor rhythms. All the signal controls are listed in Table 2, along with some of their main features.

### 3.1. Visual Evoked Potentials (VEPs)

VEPs are brain activity modulations that occur in the visual cortex after receiving a visual stimulus [101]. These modulations are relatively easy to detect since the amplitude of VEPs increases enormously as the stimulus is moved closer to the central visual field [102].

VEPs may be classified according to three different criteria [103]: (i) by the morphology of the optical stimuli, (ii) by the frequency of visual stimulation; and (iii) by field stimulation. According to the first criterion, VEPs may be caused by using flash stimulation or using graphic patterns such as checkerboard lattice, gate, and random-dot map. According to the frequency, VEPs can also be classified as transient VEPs (TVEPs) and as steady-state VEPs (SSVEPs). TVEPs occur when the frequency of visual stimulation is below 6 Hz, while SSVEPs occur in reaction to stimuli of a higher frequency [101,104]. Lastly, according to the third criterion, VEPs can be divided into whole field VEPs, half field VEPs, and part field VEPs depending on the area of on-screen stimulus. For instance, if only half of the screen displays graphics, the other half will not display any visual stimulation, and the person will look at the centre of the screen, which will induce a half field VEP.

TVEPs can be elicited by any change in the visual field. Those used most frequently are TVEPs are: (i) flash TVEPs that are caused by flashing lights; (ii) pattern onset/offset TVEPs that are caused by letting a pattern appear abruptly on a diffuse background; and (iii) pattern reversal TVEPs that are caused by reversing the phase of a pattern *i.e.*, a checkerboard lattice that changes the checks from black to white and from white to black abruptly [105].

The evoked responses vary with the stimulus presented. Flash TVEPs present a series of negative and positive peaks. The most prominently peaks are negative (N2) and positive (P2) peaks at around 90 ms and 120 ms respectively [105]. Pattern onset/offset TVEPs have three main peaks: C1 (positive, 75 ms), C2 (negative, 125 ms), and C3 (positive, 150 ms) [105]. Pattern reversal TVEPs usually present one negative peak at 75 ms, one positive peak at 100 ms, and one negative peak at 135 ms [105].

SSVEPs are elicited by the same visual stimulus. In this case, the stimulus changes at a frequency higher than 6 Hz. If the stimulus is a flash, SSVEP shows a sinusoidal-like waveform, the fundamental frequency of which is the same as the blinking frequency of the stimulus. If the stimulus is a pattern, the SSVEP occurs at the reversal rate and at their harmonics [106]. In contrast to TVEP, constituent discrete frequency components of SSVEPs remain closely constant in amplitude and phase over long periods of time [107]. SSVEPs are less susceptible than TVEPs to artifacts produced by blinks and eye movements [108] and to electromyographic noise contamination [109]. Indeed, TVEPs not are typically used for BCI.

SSVEP-based BCIs allow users to select a target by means of an eye-gaze. The user visually fixes attention on a target and the BCI identifies the target through SSVEP features analysis. Considering a BCI as a communications channel, SSVEP-based BCIs can be classified into three categories depending on the specific stimulus sequence modulation in use [110]: time modulated VEP (t-VEP) BCIs, frequency modulated VEP (f-VEP) BCIs, and pseudorandom code modulated VEP (c-VEP) BCIs. VEPs that react to different stimulus sequences should be orthogonal or near orthogonal to each other in some domain to ensure reliable identification of the target [110]. In a t-VEP BCI, the flash sequences of different targets are orthogonal in time. That is, the flash sequences for different targets are either strictly non-overlapping or stochastic. In an f-VEP BCI, each target is flashed at a unique frequency, generating a periodic sequence of evoked responses with the same fundamental frequency as its harmonics. In a c-VEP BCI, pseudo-random sequences are used. The duration of ON and OFF states of each target’s flash is determined by a pseudorandom sequence. Signal modulations can optimize the information transfer rate. Indeed, code modulation provides the highest communication speed. Table 3 summarizes the features of each modulation.

The typical VEP-based BCI application displays flashing stimuli, such as digits or letters, on a screen to induce SSVEPs while the user stares at one of the symbols. The user can move their gaze to the flashing digits or letters, in order to communicate with the computer [111]. The advantage of this type of control signal is that very little training is required. However, it presents the drawback that the user has to watch the screen and keep his eyes fixed on one point. This type of control signal can only be used for exogenous BCIs (see Section 0). Therefore, VEPs are not suitable for patients in advanced stages of Amyotrophic Lateral Sclerosis (ALS) or with uncontrollable eye or neck movements. Some independent SSVEP-based BCIs that are controlled by the attention of the user have been introduced to overcome this drawback [112,113].

SSVEP are usually elicited through light-emitting diodes (LEDs), cathode-ray tube (CRT) monitors, or liquid crystal display (LCD). LEDs outperform LCD or CRT stimulators but they need more complex hardware. LCD and CRT monitors make the target presentation easier than LED stimulators, because both systems can easily be connected to a PC. However, LED stimulators may be preferable for a multiple target BCI, because the refresh rate of an LCD or CRT monitor can limit the number of targets. LED stimulators offer more versatility because the flickering frequency and phase of each LED can be controlled independently by a programmable logic device [114]. The stimulation decision can be made on the basis of the number of choices that the BCI offers [115]. LCD screens are optimal for low complexity BCI (less than 10 choices), because they induce less eye-tiredness than CRT screens. For medium complexity BCI (10–20 choices), LCD or CRT screens are optimal. For high complexity BCI (more than 20 commands), LED are preferred.

### 3.2. Slow Cortical Potentials (SCPs)

SCPs are slow voltage shifts in the EEG that last a second to several seconds. SCPs belong to the part of the EEG signals below 1 Hz [116]. SCPs are associated with changes in the level of cortical activity. Negative SCPs correlate with increased neuronal activity, whereas positive SCPs coincide with decreased activity in individual cells [116]. These brain signals can be self-regulated by both healthy users and paralyzed patients to control external devices by means of a BCI. SCP shifts can be used to move a cursor and select the targets presented on a computer screen [117].

People can be trained to generate voluntary SCP changes using a thought-translation device [117]. The thought-translation device is a tool used for self-regulation SCP training, which shows visual-auditory marks so that the user can learn to shift the SCP. The thought-translation device typically comprises a cursor on a screen in such a way that the vertical position of the cursor constantly reflects the amplitude of SCP shifts. Although most thought-translation devices show continuous feedback, it is possible to train SCP self-modulation in the absence of continuous feedback [118].

Success in SCP self-regulation training depends on numerous factors, such as the patient’s psychological and physical state, motivation, social context, or the trainer-patient relationship [117]. It is known that the learning capability of the user drastically affects SCP modulation training. Self-regulation training is therefore strongly recommended for patients at the early stage of a progressive disease [117]. Furthermore, initial SCP modulation skills have an effect on future performance following training [119]. Therefore, the value of SCPs as a suitable control signal for each patient can only be determined on the basis of initial trials. Other factors, such as sleep quality, pain, and mood also have an influence on self-regulation performance [117]. Their effects are not identical for all patients and further investigation is certainly needed to establish general rules on this matter.

Self-regulation of SCPs has been tested extensively with patients suffering from ALS [120–122]. Typical accuracy rates achieved for SCP classification are acceptable and vary between 70 and 80 per cent, but the rates of information provided by SCP-based BCI are relatively low. Besides, longer training is required to use SCP-based BCI and it is likely that users will need continuous practice for several months.

### 3.3. P300 Evoked Potentials

P300 evoked potentials are positive peaks in the EEG due to infrequent auditory, visual, or somatosensory stimuli. These endogenic P300 responses are elicited about 300 ms after attending to an oddball stimulus among several frequent stimuli [123,124]. Some studies have proven that the less probable the stimulus, the larger the amplitude of the response peak [125]. The use of P300-based BCIs does not require training. However, the performance may be reduced because the user gets used to the infrequent stimulus and consequently P300 amplitude is decreased [126].

A typical application of a BCI based on visual P300 evoked potentials comprises a matrix of letters, numbers, or other symbols or commands [123,127,128]. The rows or columns of this matrix are flashed at random while the EEG is monitored. The user gazes at the desired symbol and counts how many times the row or column containing the desired choice flashes. P300 is elicited only when the desired row or column flashes. Thus, the BCI uses this effect to determine the target symbol. Due to the low signal-to-noise ratio in EEG signals, the detection of target symbols from a single trial is very difficult. The rows or columns must be flashed several times for each choice. The epochs corresponding to each row or column are averaged over the trials, in order to improve their accuracy. However, these repetitions decrease the number of choices per minute, e.g., with 15 repetitions, only two characters are spelled per minute [123]. Although most of the applications based on P300 evoked potentials employ visual stimuli, auditory stimuli have been used for people with visual impairment [129].

P300-based BCIs provide a very low rate of information transmission because the classifier based on an average is too simple, and the accuracy of P300 potential detection is too low [130]. Consequently, too many trials are required to select a single symbol in the matrix. Accuracy of P300-based BCIs can be improved, while using a more complicated classifier than a simple average to ensure that the number of repetitions remain unaffected [130,131]. Other studies have proven that the detection accuracy of visual P300 evoked potentials also depends on the properties of the visual matrix such as the dimensions or colors of the symbols. Performance decreases when matrices with smaller symbols are used [132], and it is enhanced when a green and blue chromatic flicker matrix is used, rather than a gray and black one [133,134].

Information transmission rates provided by P300-based BCI can be also improved by considering the BCI as a noisy transmission system. BCI can therefore benefit from the use of error correcting codes [135]. However, optimizing the code solely according to the maximal minimum-Hamming-distance implies an increase in target frequency of target stimuli which might violate physiological constraints leading to difficulties in classifying the individual ERPs, due to overlap and refractory effects. Further, overlap and refractory effects are generally the main error source in these kinds of BCIs [136]. Some recent novel approaches have tried to reduce them, by superimposing the targets on a checkerboard [137] or by using alternative stimulus type methods based on motion [136].

The P300 response is not markedly affected by whether or not the subject gazes directly at the target, in contrast to the VEP response, which is larger when the target is foveated [138]. This distinction is important for clinical applications, because eye movements are often impaired or lost in the target population. Nevertheless, the performance of a P300-based BCI is substantially improved when subjects gaze at the desired item [138]. Therefore, the performance of the visual P300-based BCIs depends not only on the P300-evoked potential, but also on the VEP response that, in turn, strongly depends on eye-gaze direction.

### 3.4. Sensorimotor Rhythms (mu and beta rhythms)

Sensorimotor rhythms comprise mu and beta rhythms, which are oscillations in the brain activity localized in the mu band (7–13 Hz), also known as the Rolandic band, and beta band (13–30 Hz), respectively. Both rhythms are associated in such a way that some beta rhythms are harmonic mu rhythms, although some beta rhythms may also be independent [139]. The amplitude of the sensorimotor rhythms varies when cerebral activity is related to any motor task although actual movement is not required to modulate the amplitude of sensorimotor rhythms [140,141]. Similar modulation patterns in the motor rhythms are produced as a result of mental rehearsal of a motor act without any overt motor output [140]. Sensorimotor rhythms have been used to control BCIs, because people can learn to generate these modulations voluntarily in the sensorimotor rhythms [36,142].

**Figure 2.**
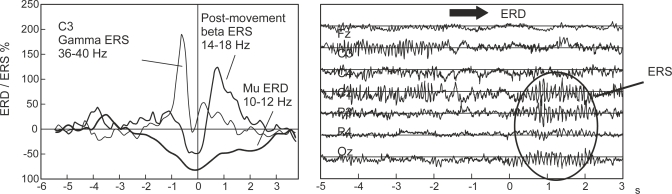
**Left panel**: Superimposed band power time courses computed for three different frequency bands (10–12 Hz, 14–18 Hz, and 36–40 Hz) from EEG trials recorded from electrode position C3 during right index finger lifting. EEG data triggered with respect to movement-offset (vertical line at t = 0 s); **Right panel**: Examples of ongoing EEG recorded during right finger movement (adapted from [36]).

Sensorimotor rhythms can endure two kinds of amplitude modulations known as event-related desynchronization (ERD) and event-related synchronization (ERS) that are generated sensory stimulation, motor behavior, and mental imagery [36]. ERD involves an amplitude suppression of the rhythm and ERS implies amplitude enhancement. Figure 2 (left panel) shows the temporal behavior of ERD and ERS during a voluntary movement experiment which involves brisk finger lifting [36]. The mu band ERD starts 2.5 s before movement on-set, reaches the maximal ERD shortly after movement-onset, and recovers its original level within a few seconds. In contrast, the beta rhythm shows a short ERD during the movement initiation of movement, followed by ERS that reaches the maximum after movement execution. This ERS occurs while the mu rhythm is still attenuated. Figure 2 also shows the gamma oscillation (36–40 Hz), which is another rhythm related to motor tasks as well [36]. Gamma rhythms reveal an ERS shortly before movement-onset. Finally, the right panel of Figure 2 illustrates that simultaneous ERD and ERS are possible at different scalp locations [36].

Sensorimotor rhythms are related to motor imagery without any actual movement [141]. This makes it possible to use sensorimotor rhythms for the design of endogenous BCIs, which are more useful than exogenous BCIs. Nevertheless, self-control of sensorimotor rhythms is not easy, and most people have difficulties with motor imagery. People tend to imagine visual images of related real movements, which is not sufficiently useful for a BCI system, because the patterns of these sensorimotor rhythms differ from actual motor imagery. User training should emphasize kinesthetic experiences instead of visual representations of actions [143]. Motor imagery training is traditionally based on visual or auditory feedback [144]. This kind of training asks the users to perform a certain motor imagery task, and then the sensorimotor rhythms are extracted and classified by comparing them with a reference. Finally, visual or auditory feedback is provided to the participant according to the success of the result. This kind of training has been widely used although usually its effectiveness was not very high [145]. Hwang *et al.* [145] presented more effective motor imagery training based on a system that displayed real-time cortical activity as feedback, which allowed the users to watch their own cortical activity through a real-time monitoring system.

Sensorimotor rhythms have been investigated extensively in BCI research. Well-known BCI systems such as Wadsworth [146], Berlin [147], or Graz [148] BCIs employ sensorimotor rhythms as control signals. The BCIs based on sensorimotor rhythms can operate in either synchronous or asynchronous mode. The latest advances in the field of BCIs based on sensorimotor rhythms have shown that it is possible to predict human voluntary movements before they occur based on the modulations in sensorimotor rhythms [149]. Furthermore, this prediction could be provided without the user making any movements at all.

## 4. Types of BCIs

The BCIs can be categorized into (i) exogenous or endogenous and (ii) synchronous (cue-paced) or asynchronous (self-paced). Types of BCI are listed in Tables 4 and 5, along with information related to brain signals that can be modulated to convey information as well as advantages and disadvantages. Also, BCIs can be classified into dependent and independent [2]. This distinction will not be detailed in this review because it is very similar to exogenous and endogenous distinction. Advantages and disadvantages in both taxonomies are analogous.

According to the nature of the signals used as input, BCI systems can be classified as either exogenous or endogenous. Exogenous BCI uses the neuron activity elicited in the brain by an external stimulus such as VEPs or auditory evoked potentials [150]. Exogenous systems do not require extensive training since their control signals, SSVEPs and P300, can be easily and quickly set-up. Besides, the signal controls can be realized with only one EEG channel and can achieve a high information transfer rate of up to 60 bits/min. On the other hand, endogenous BCI is based on self-regulation of brain rhythms and potentials without external stimuli [150]. Through neurofeedback training, the users learn to generate specific brain patterns which may be decoded by the BCI such as modulations in the sensorimotor rhythms [151] or the SCPs [117]. The advantage of an endogenous BCI is that the user can operate the BCI at free will and move a cursor to any point in a two-dimensional space, while an exogenous BCI may constrain the user to the choices presented. Also, endogenous BCI are especially useful for users with advanced stages of ALS or whose sensory organs are affected. Table 4 summarizes the differences between exogenous and endogenous BCIs.

According to the input data processing modality, BCI systems can be classified as synchronous or asynchronous. Synchronous BCIs analyze brain signals during predefined time windows. Any brain signal outside the predefined window is ignored. Therefore, the user is only allowed to send commands during specific periods determined by the BCI system. For example, the standard Graz BCI [148] represents a synchronous BCI system. The advantage of a synchronous BCI system is that the onset of mental activity is known in advance and associated with a specific cue [152]. Moreover, the patients may also perform blinks and other eye movements, which would generate artifacts, if the BCI did not analyze the brain signals to avoid their misleading effects. This simplifies the design and evaluation of synchronous BCI. Asynchronous BCIs continuously analyze brain signals no matter when the user acts. They offer a more natural mode of human-machine interaction than synchronous BCI. However, asynchronous BCIs are more computation demanding and complex. Table 5 summarizes the differences between synchronous and asynchronous BCIs.

## 5. Features Extraction and Selection

Different thinking activities result in different patterns of brain signals. BCI is seen as a pattern recognition system that classifies each pattern into a class according to its features. BCI extracts some features from brain signals that reflect similarities to a certain class as well as differences from the rest of the classes. The features are measured or derived from the properties of the signals which contain the discriminative information needed to distinguish their different types.

The design of a suitable set of features is a challenging issue. The information of interest in brain signals is hidden in a highly noisy environment, and brain signals comprise a large number of simultaneous sources. A signal that may be of interest could be overlapped in time and space by multiple signals from different brain tasks. For that reason, in many cases, it is not enough to use simple methods such as a band pass filter to extract the desired band power.

Brain signals can be measured through multiples channels. Not all information provided by the measured channels is generally relevant for understanding the underlying phenomena of interest. Dimension reduction techniques such as principal component analysis or independent component analysis can be applied to reduce the dimension of the original data, removing the irrelevant and redundant information. Computational costs are thereby reduced.

Brain signals are inherently non-stationary. Time information about when a certain feature occurs should be obtained. Some approaches divide the signals into short segments and the parameters can be estimated from each segment. However, the segment length affects the accuracy of estimated features. FFT performs very poorly with short data segments [153]. Wavelet transform or adaptive autoregressive components are preferred to reveal the non-stationary time variations of brain signals. Also, a novel technique called stationary subspace analysis (SSA) has recently been introduced to deal with the non-stationarity of EEG signals [154]. SSA decomposes multivariate time series into stationary and non-stationary components.

Multiples features can be extracted from several channels and from several time segments before being concatenated into a single feature vector. One of the major difficulties in BCI design is choosing relevant features from the vast number of possible features. High dimensional feature vectors are not desirable due to the “curse of dimensionality” in training classification algorithms (see next section). The feature selection may be attempted examining all possible subsets of the features. However, the number of possibilities grows exponentially, making an exhaustive search impractical for even a moderate number of features. Some more efficient optimization algorithms can be applied with the aim of minimizing the number of features while maximizing the classification performance.

This section discusses methods to obtain the relevant characteristics of brain signals as well as feature selection methods. Firstly, dimensional reduction methods, such as principal component analysis or independent component analysis are explained. Secondly, time and/or frequency methods, such as matched filtering or wavelet transform, and parametric modeling, such as autoregressive component, are also surveyed. Thirdly, an explanation is given of the common spatial pattern algorithm. This method designs a preprocessing spatial filter, by means of spatial covariance from input data and signal whitening, that enhances the difference between classes before the feature extraction stage. And, finally, feature selection methods such as genetic algorithms or sequential selection are included. All these methods, including feature extraction and feature selection methods, are listed respectively in Tables 6 and 7 along with information on their properties and BCI applications.

### 5.1. Principal Component Analysis (PCA)

PCA is a statistical features extraction method that uses a linear transformation to convert a set of observations possibly correlated into a set of uncorrelated variables called principal components. Linear transformation generates a set of components from the input data, sorted according to their variance in such a way that the first principal component has the highest possible variance. This variance allows PCA to separate the brain signal into different components.

PCA projects the input data on a *k*-dimension eigenspace of *k* eigenvectors, which are calculated from the covariance matrix Σ of the training data *p =* [*p_1_* *p_2_* *^⋯^* *p_n_*] [155]. *p_i_* is *i*-th *d*-dimension training sample, and *n* is the number of samples.

The covariance matrix Σ is computed as:

(1) $$\Sigma =\sum_{i=1}^{n}(p_{i}-m){\left(p_{i}-m\right)}^{t}$$

where, $m=\frac{1}{n}\sum_{i=1}^{n}p_{i}$ is the mean vector of the training samples p_i_.

The covariance matrix Σ is a real and symmetric *d x d* matrix, therefore Σ has *d* different eigenvectors and eigenvalues. By means of the eigenvalues, it is possible to know which eigenvectors represent the most significant information contained in the dataset. The eigenvectors with the highest eigenvalue represent the principal components of the training dataset *p*. PCA selects that *k,* with *k < d*, eigenvectors having the largest eigenvalues. These selected eigenvectors serve to build a projection matrix ***A*** that will be used to extract the feature vector from the test data *q*. The *k* eigenvectors are sorted into columns in Matrix ***A***, such that the first column of ***A*** corresponds to the largest eigenvalue. Finally, PCA computes the feature vector *v* from the data in matrix ***A***, by projecting the test data *q* onto the new subspace, such that:

(2) $$v=A^{t}(q-m)$$

where, $m=\frac{1}{n}\sum_{i=1}^{n}p_{i}$ represents the mean vector of training samples *p_i_*.

PCA is also a procedure to reduce the dimension of the feature. Since the number of columns is less than the number of eigenvectors, the dimension of the output projected data is less than the dimension of the input data. This decrease in dimensionality can reduce the complexity of the subsequent classifying step in a BCI system.

PCA does not always guarantee a good classification since the best discriminating components may not figure among the largest principal components [156]. PCA reduces data dimension by seeking a new optimal representation of data in terms of minimal mean-square-error between the representation and the original data. It will not guarantee that the discriminative features are optimal for classification. Despite this shortcoming, it has been proven that PCA is a reliable noise reduction method.

With regard to the applications of PCA in BCI systems, PCA has been used to identify the artifactual components in a reasonably successful way in EEG signals and to reconstruct the signals without the artifactual components [157,158]. Nevertheless, the artifacts must not be correlated with the EEG signal for PCA to function in this way. PCA has also been employed, in order to reduce feature space dimensionality [155].

### 5.2. Independent Component Analysis (ICA)

ICA is a statistical procedure that splits a set of mixed signals into its sources with no previous information on the nature of the signal. The only assumption involved in ICA is that the unknown underlying sources are mutually independent in statistical terms. ICA assumes that the observed EEG signal is a mixture of several independent source signals coming from multiple cognitive activities or artifacts. ICA therefore expresses the resulting EEG signal ***x****(t)* in relation to their sources ***s****(t)* as:

(3) x(t)=f(s(t))+n(t)

where, ***f*** is any unknown mixer function, and ***n****(t)* is an additive random noisy vector. The dimension of the input vector ***s****(t)* depends on the number of sources. The dimension of output vector ***x****(t)* is equal to the number of measured data channels. The number of sources is usually assumed to be less than or equal to the number of channels although more generalized ICA methods are possible [159].

The whole ICA problem consists in the calculation of the unmixing function by inverting ***f*** and obtaining an estimation of ***s****(t)*, by mapping ***x****(t)* to the source space. To solve the problem, ICA can fall into two different models on the basis of ***f***, which may be either a linear or nonlinear function. The nonlinear assumption is suitable in those cases where the linear model might be too simple to describe the observed data ***x****(t)*. However, the nonlinear problem is usually too complex and generally intractable due to its high number of indeterminations. The assumption of a linear mixing function simplifies Equation (3). It is possible to rewrite it as a matrix multiplication where ***A*** is the mixing matrix. The Equation (4) gives the mathematical expression of the linear ICA model:

(4) x(t)=As(t)+n(t)

Although the approximation given by Equation (4) can be considered too simple, it works reasonably well in brain signal processing applications. Furthermore, it is possible to remove the noise term ***n****(t)* from Equation (4), by assuming that the observed data is noiseless or that the noise is too weak for consideration [160,161]. Finally, ***s****(t)* and ***A*** are obtained from ***x****(t)* by means of certain algorithms, such as Infomax [162] or further modification of the Infomax [163].

ICA has traditionally been used as a preprocessing tool before the feature extraction step, in order to remove ocular artifacts in BCI systems [164–166]. Although ICA has been proven to be a powerful and robust tool for artifact removal in signal analysis, some studies have indicated that artifact suppression may also corrupt the power spectrum of the underlying neural activity [167]. In addition, ICA requires that the artifacts are independent in relation to the EEG signal.

It is also possible to find authors that have employed ICA as a classifier. ICA can be modified to classify EEG signals by fitting the generative ICA model to each task and employing Bayes’ rule to create the classifier [168].

### 5.3. AutoRegressive Components (AR)

AR spectral estimation is a method for modeling signals. AR models the EEG signal as the output random signal of a linear time invariant filter, where the input is white noise with a mean of zero and a certain variance of σ^2^. The aim of the AR procedure is to obtain the filter coefficients, since it is assumed that different thinking activities will produce different filter coefficients. The filter coefficients will be used as the features of the signal.

AR assumes that the transfer function of the filter will only contain poles in the denominator. The number of poles in the denominator corresponds to the order of the autoregressive model. The assumption of an all-pole filter makes the filter coefficients computation easier because it is only necessary to solve linear equations.

Mathematically, the AR model of order *p* describes the EEG signal *y(t)* as:

(5) $$y(t)=a_{1}y(t-1)+a_{2}y(t-2)+a_{3}y(t-3)+\cdots +a_{p}y(t-p)+n(t)$$

where, *a_i_* is the *i-th* filter coefficient, and *n(t)* is the noise. There are several methods that compute the filter coefficients such as the Yule-Walker, Burg, covariance, and forward-backward algorithms [169]. The resulting coefficients can be used to estimate the power spectrum of the EEG signal *y(ω)*, such that:

(6) $$y(\omega )=\frac{1}{{\left|1-\sum_{k=1}^{p}a_{k}e^{-jk\omega }\right|}^{2}}$$

where, *a_k_* are the estimated filter coefficients, and *p* is the AR model order, in other words, the number of poles.

In the AR model, the determination of an appropriate order *p* for a given input signal is a trade-off issue. If the order is too low to model the input signal, the result will not faithfully represent the signal because the spectrum is too smooth. In contrast, if the order is too high, the spectrum may exhibit spurious peaks.

AR spectral estimation is preferred to Fourier Transform, because of its superior resolution for short time segments [170]. Nevertheless, AR performs poorly when the signal is not stationary [171]. Due to the non-stationary nature of EEG signals, a multivariate adaptive AR (MVAAR) model has been proposed to design more effective on-line BCI systems. Jiang *et al.* [172] applied MVAAR for the classification of motor imagery, showing that MVAAR is a valuable adaptive method for feature extraction. The computation algorithm was very similar to the original AR model. In a BCI with *m* channels, the vector of *m* EEG values, at each point in time *k*, was represented as:

(7) $${\vec{y}}_{k}={\left[y_{k,1}\;\;y_{k,2}\cdots y_{k,m}\right]}^{T}$$

As in the AR case, the MVAAR model was expressed as:

(8) $${\vec{y}}_{k}=A_{1}{\vec{y}}_{k-1}+A_{2}{\vec{y}}_{k-2}+A_{3}{\vec{y}}_{k-3}+\cdots +A_{p}{\vec{y}}_{k-p}+{\vec{n}}_{k}$$

where, *n⃗_k_* was the vector of white noise values, *A*_1_ ⋯ *A_p_* were the adaptive coefficients, and *p* was the model order. The Recursive Least Squares algorithm, a special variant of the Kalman Filter, were used to update coefficients *A*_1_ ⋯ *A_p_* at every point *k*.

### 5.4. Matched Filtering (MF)

MF is a feature extraction method that attempts to detect a specific pattern on the basis of its matches with predetermined known signals or templates. The intention of the user is revealed by means of the correlation between the unknown EEG signals and the set of templates. Each template represents an intention of the user. A higher correlation would imply better matching between the template and the user’s intention. Each matched filter can simply be modeled as a sum of the harmonically related sinusoidal components [151]:

(9) $$\mathrm{MF}(n)=\sum_{k=1}^{N}a_{k}\;\text{cos}\;\left(\frac{2\pi kf_{F}}{f_{s}}n+\Phi_{k}\right)$$

where, *n* is the template sample number, *f_s_* is the sampling frequency, *f_F_* is the fundamental frequency of the rhythm template, *N−1* is the number of harmonics, and *a_t_* and *Φ_k_* are the amplitude and phase of the individual harmonics, respectively. The model parameters *a_t_* and *Φ_k_* can be obtained from the FFT spectrum [151].

MF has been proven especially effective for the detection of waveforms with consistent temporal characteristics. Krusienski *et al.* [151] used MF for the identification of user intentions through μ-rhythms and Brunner *et al.* [173] also used it for SSVEP feature extraction.

### 5.5. Wavelet Transform (WT)

WT is a mathematical tool widely used for extracting information from many different kinds of data, such as audio or image data, among others. WT is particularly suitable when signals are not stationary, because it provides a flexible way of representing the time-frequency of a signal [174].

Wavelets are functions of varying frequency and limited duration that allow simultaneous study of the signal in both the time and the frequency domain [175], in contrast to other modalities of signal analysis such as Fourier transform (FT). FT provides only an analysis of the signal activity in the frequency domain. FT gives information about the frequency content, but it is not accompanied by information on when those frequencies occur. Short-term Fourier Transform (STFT) was proposed to overcome this shortcoming of the Fourier analysis. The STFT divides the signal into successive time windows and applies the FT in each epoch of the signal in time. In this approach, the design of window length is a trade-off because smaller windows lead to higher temporal resolution but also to lower frequency resolution at the same time. The WT overcomes this drawback by decomposing the signal in both the time and the frequency domain at multiple resolutions, by using a modulated window that is shifted along the signal at various scales.

Continuous wavelet transform (CWT) is defined as the convolution of the signal *x*(*t*) with the wavelet function *ψ_s,τ_*(*t*) [175]:

(10) $$w\;(s,\tau )=\int_{-\infty }^{\infty }x(t)\psi_{s,\tau }^{*}\;(t)\mathrm{dt}$$

*w*(*s*, *τ*) is the wavelet coefficient that corresponds to the frequency associated with the scale *s* and the time *τ* of the wavelet function *ψ*_*s*, τ_(*t*), and the symbol ‘*’ expresses the complex conjugation. The wavelet function *ψ*_*s*,*τ*_(*t*) is a dilated and shifted version of a *mother wavelet ψ*(*t*):

(11) $$\psi_{s,\tau }\;(t)=\frac{1}{\sqrt{s}}\psi (\frac{t-\tau }{s})$$

A mother wavelet can take multiples shapes, but it always satisfies the next condition:

(12) $$\int_{-\infty }^{\infty }\psi (t)dt=0$$

The CWT defined in the Equation (10) is actually a kind of template matching, similar to a matched filter in which the cross variance between the signal and a predefined waveform is calculated [151]. The advantage of the CWT over classic template matching methods arises from the special properties of the wavelet template. The wavelets are suitable for transient signal analysis, in which the spectral properties of the signal vary over time [176].

WT is a powerful tool for the decomposition of transient brain signals into their constituent parts, based on a combination of criteria such as frequency and temporal position. Signals of identical frequency ranges can be distinguished by means of the temporal position. Likewise, it is possible to separate temporally overlapping processes thanks to the different frequency content.

The CWT introduces a lot of redundancy and complexity since it involves the analysis of a signal at a very high number of frequencies using multiple dilations and shifting of the mother wavelet. Discrete wavelet transform (DWT) was introduced to reduce this redundancy and complexity. The DWT translates and dilates the mother wavelet in certain discrete values only [177]. Farina *et al.* [178] showed a pattern recognition approach for the classification of single trial movement-related cortical potentials, where the feature space is built from coefficients of a discrete wavelet transformation. Although DWT is less redundant and less complex than CWT, CWT is still employed to extract features from P300 and SCP, because it can clarify subtle information that DWT is unable to extract [179].

The use of WT requires the selection of a mother wavelet. Many different mother wavelets can be found in BCI applications and the selection of any one depends on what types of features need to be extracted from the signal The Mexican Hat wavelet is well localized in the time domain and is employed for the localization of ERP components in time [179]. The Morlet wavelet is well localized in the frequency domain and has been used for the analysis of gamma activity [180]. The bi-scale wavelet has been employed successfully for designing an asynchronous BCI based on detection of imaginary movement in the 1–4 Hz frequency range [181]. Also, the Daubechies wavelet, a very well-known mother wavelet, has been used for the classification of SCPs [182].

### 5.6. Common Spatial Pattern (CSP)

CSP is a feature extraction method that projects multichannel EEG signals into a subspace, where the differences between classes are highlighted and the similarities are minimized. It aims to make the subsequent classification much more effective, by designing a spatial filter that transforms the input data into output data with an optimal variance for the subsequent discrimination [183]. CSP has been designed for the analysis of multichannel data belonging to 2-class problems. Nevertheless, some extensions for multiclass BCIs have also been proposed [184].

CSP calculates the normalized spatial covariance C from the input data E, which represents the raw data of a single trial, by means of:

(13) $$C=\frac{EE^{\prime }}{\mathrm{trace}(EE^{\prime })}$$

where, *E* is an *N x T* matrix, in which T is the number of channels, *i.e.*, recording electrodes, and N the number of samples per channel. The apostrophe ′ denotes the transpose operator, while *trace(x)* is the sum of the diagonal elements of *x*.

Assuming CSP is used to classify two classes, e.g., left and right motor imagery, CSP calculates the spatial covariances *C̅_l_* and *C̄_r_* for each of the two classes by averaging the covariances over the successive training trials of each class over time. The composite spatial covariance *C_c_* is computed as:

(14) $$C_{c}=\bar{C_{l}}+{\bar{C}}_{r}$$

Since *C_c_* is real and symmetric, it can be factored as *C_c_* *= U_c_λ_c_U′_c_*, where *U_c_* is the matrix of eigenvectors, and *λ_c_* is the diagonal matrix of eigenvalues.

By means of the whitening transform:

(15) $$P=\sqrt{\lambda_{c}^{-1}}U_{c}^{\prime }$$

the variances are equalized in the space spanned by *U′_c_* and all eigenvalues of *PC̄cP*′ are equal to one. If *C̄_l_* and *C̄r* are transformed as:

(16) $$S_{l}=P{\bar{C}}_{l}P^{\prime }$$

(17) $$S_{r}=P{\bar{C}}_{r}P^{\prime }$$

then, *S_l_* and *S_r_* will share common eigenvectors. If *S_l_* *= Bλ_l_B*′, then *S_r_ = Bλ_r_B*′, and *λ_l_* *+ λ_r_* *= I*, where *I* is the identity matrix. As a result of the sum of two corresponding eigenvalues being always one, the eigenvectors with the largest eigenvalues for *S_l_* correspond to the smallest eigenvalue for *S_r_*, and vice versa. This property is very useful for subsequent classification, because the variance of the signal is maximized for one class while minimized for the other class.

Finally, the feature vector Z is obtained from the trial E as:

(18) Z=WE

where, *W* = (*B*′*P*)′ is the spatial filter matrix built by the CSP procedure.

CSP increases the accuracy of synchronous BCIs where it is allowed to send signals only during certain predefined time periods. However, CSP does not offer the same improvement in asynchronous BCIs. This is mainly due to the nonstationary properties of EEG signals [185]. Also, the performance of CSP is affected by the spatial resolution, and it has been proven that some electrode locations offer more discriminative information for some specific brain activities than others. For these reasons, several methods improving the original CSP method have been proposed to increase the performance: Wavelet Common Spatial Pattern (WCSP) [185], Common Spatio-Spectral Pattern (CSSP) [186], and Common Sparse Spectral Spatial Pattern (CSSSP) [187].

### 5.7. Genetic Algorithm (GA)

GA is an optimization procedure to establish whether a certain set of features is the most efficient. GA has been used in very diverse fields to solve optimization problems. In BCI research, GA has been used as an automatic method to extract an optimal set of relevant features [188,189].

**Figure 3.**
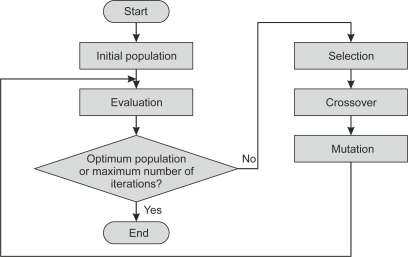
Genetic algorithm.

The baseline of the algorithm is a population of candidate solutions called individuals, creatures, or phenotypes which are encoded by strings named chromosomes or the genotype of the genome. These strings are coded either by binary information or no binary information. The standard steps of the GA can be explained briefly as follows (Figure 3). GA begins with an initial population which is randomly generated unless the algorithm has previous of the final solution. In the case of having initial information, the initial population may be directed towards areas where optimal solutions are more likely to reduce the number of iterations. The fitness of every individual population is evaluated. According to their fitness, some representatives of the population may be discarded to vacate space for newly generated individuals. Other individuals may be selected as parents in order to breed new individuals. Also, some individuals may be stochastically selected to keep diversity in the population preventing premature convergence. After the selection step, the individuals are crossed with each other. In the crossover step, mating is performed among the selected parents to generate one or more offspring. To keep a fixed population size, the number of offspring is usually the same as the number of discarded individuals. The parents’ genes are split into pieces and then combined to form new offspring. Following the crossover step, mutations are introduced to alter the population in order to avoid converging towards a local suboptimum solution before exploring the entire search space. As a result of the mutation, it is possible to discover areas that cannot be explored by crossover. Finally, the fitness of the new population is evaluated. When an acceptable solution is reached or the maximum number of generations has been produced, the algorithm is terminated. Otherwise, another iteration of the algorithm is produced.

### 5.8. Sequential Selection

Sequential selection is an optimization approach that aims at finding the optimal subset of features by adding or removing features sequentially. There are two algorithms that perform sequential selection: *sequential forward selection* and *sequential backward selection.*

*Sequential forward selection* (SFS) [190] is a bottom up algorithm. Firstly, the best individual feature is found as the first feature in the subset. Next, for each subsequent step, the algorithm chooses the feature from the remaining set, which in combination with the previously selected features, yields the best subset of features. Finally, the algorithm finishes when the required number of features is reached. The shortcoming of this algorithm is that the superfluous features are not removed once other features are added. *Sequential backward selection* (SBS) [190], in contrast to SFS*,* is a top down process. The process starts with the entire set of features and removes step by step features in such a way that the error is as low as possible. This algorithm is also suboptimal, because it discards some features that may be helpful after discarding other features. SFS has been used with success in the field of BCIs [191,192].

Another refined method is introduced to partially overcome the aforementioned deficiencies. This method, known as *plus l take away r method* (*l > r*), adds *l* features, and remove *r* features that is not working well with other selected features. *Sequential forward floating search* (SFFS) or *sequential backward floating search* (SBFS) are based on the *plus l-take away r* method [193]. SFFS starts with a null feature set and, for each step, the *r* best features are included in the current feature set. In other words, *r* steps of SFS are performed. Next, the algorithm verifies the possibility that some feature may be excluded. Then, *l* worst features are eliminated from the set; in other words, *l* steps of SBS. SFFS increases and decreases the number of features until the desired number of features is reached. SBFS works analogously, but starting with the full feature set and performing the search until the desired dimension is reached, using SBS and SFS steps.

In BCI research, SFFS has been used to reduce the dimensionality of the feature space to an appropriate size for the available training data [194–196].

## 6. Artifacts in BCIs

Artifacts are undesirable signals that contaminate brain activity and are mostly of non-cerebral origin. Since the shape of neurological phenomenon is affected, artifacts may reduce the performance of BCI-based systems. Artifacts may be classified into two major categories: physiological artifacts and non-physiological or technical artifacts.

Physiological artifacts are usually due to muscular, ocular and heart activity, known as electromyography (EMG), electrooculography (EOG), and electrocardiography (ECG) artifacts respectively [197]. EMG artifacts, which imply typically large disturbances in brain signals, come from electrical activity caused by muscle contractions, which occur when patients are talking, chewing or swallowing. EOG artifacts are produced by blinking and other eye movements. Blinking makes generally high-amplitude patterns over brain signals in contrast to eye movements which produce low-frequency patterns. These electrical patterns are due to the potential difference between the cornea and the retina, as their respective charges are positive and negative. For that reason, the electric field around the eye changes when this dipole moves. EOG artifacts mostly affect the frontal area, because they are approximately attenuated according to the square of the distance [198]. Finally, ECG artifacts, which reflect heart activity, introduce a rhythmic signal into brain activity [197].

Technical artifacts are mainly attributed to power-line noises or changes in electrode impedances, which can usually be avoided by proper filtering or shielding [197]. Therefore, the BCI community focuses principally on physiological artifacts, given that their reduction during brain activity acquisition is a much more challenging issue than non-physiological artifact handling.

Several ways of handling physiological artifacts can be found in the literature. Artifacts may be avoided, rejected or removed from recordings of brain signals. Artifact avoidance involves asking patients to avoid blinking or moving their body during the experiments [199]. This approach to artifact handling is very simple, because it does not require any computation as brain signals are not assumed to have artifacts. However, this assumption is not always feasible given that some artifacts - involuntary heart beats, eye and bodily twitches- are not easily avoidable during data recording, especially in cases of strong neurological disorders [199]. Artifact rejection approaches suggest discarding the epochs contaminated by the artifacts. Manual artifact rejection is an option to remove artifacts in brain signals and an expert could identify and eliminate all artifact-contaminated epochs. The main disadvantage in using manual rejection is that it requires intensive human labor, so this approach is not suitable for on-line BCI systems. Nevertheless, this task can be performed automatically by EMG and EOG artifact detection. If EMG and EOG signals are monitored, the brain signal samples may be removed whenever ocular or muscular activity of the arms is detected [200]. Automatic rejection is an effective way of artifact handling, but it may fail when EOG amplitudes are too small. Besides, rejection methodology means that the user loses device control when artifact contaminated signals are discarded. Instead of rejecting samples, the artifact removal approach attempts to identify and remove artifacts while keeping the neurological phenomenon intact. Common methods for removing artifacts in EEG are linear filtering, linear combination and regression, BSS and PCA [197]; some of which were discussed in Section 0.

Instead of avoided, rejected or removed artifacts from recordings of brain signals, some systems acquire and process artifacts to offer a communication path that either disabled or healthy people can use in many tasks and in different environments. This kind of system is not considered a BCI, because communication is not independent of peripheral nerves and muscles. EMG computer interface [201], human-computer interface (HCI) [202], EMG-based human-computer interface [203], EMG-Based Human-Machine Interface [204], EMG-based human-robot interface [205], muscle-computer interface (MuCI) [206], man-machine interface (MMI) [207], and biocontroller interface [208] are different terms used to name communication interfaces in the scientific literature that can employ artifact signals, among others. These systems usually have greater reliability than BCIs, but they cannot be used by severely disabled people with strong constraints in voluntary movements.

## 7. Classification Algorithms

The aim of the classification step in a BCI system is recognition of a user’s intentions on the basis of a feature vector that characterizes the brain activity provided by the feature step. Either regression or classification algorithms can be used to achieve this goal, but using classification algorithms is currently the most popular approach [209].

**Figure 4.**
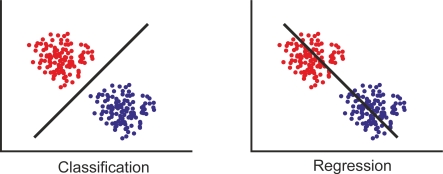
Classification and regression approaches to BCI control of two-targets (adapted from [210]). The regression algorithms employ the features extracted from EEG signals as independent variables to predict user intentions. In contrast, the classification approach uses the features extracted as independent variables to define boundaries between the different targets in feature space.

Regression algorithms employ the features extracted from EEG signals as independent variables to predict user intentions. In contrast, classification algorithms use the features extracted as independent variables to define boundaries between the different targets in feature space. McFarland *et al.* [210] illustrated the differences between the two alternatives. For a two-target case, both the regression approach and the classification approach require the parameters of a single function to be determined. In a four-target case, assuming that the targets are distributed linearly, the regression approach still requires only a single function. In contrast, the classification approach requires the determination of three functions, one for each of the three boundaries between the four targets. Therefore, the classification approach might be more useful for two-target applications and the regression approach may be preferable for greater numbers of targets, when these targets can be ordered along one or more dimensions. Moreover, the regression approach is better for continuous feedback e.g., applications which involve continuous control of cursor movement. Figure 4 illustrates the differences between classification and regression approaches.

Classification algorithms can be developed via either offline, online or both kinds of sessions. The offline session involves the examination of data sets, such as BCI competitions data sets [211], which are collected from an adaptive or closed-loop system. The statistics of the data may be estimated from observations across entire sessions and long-term computations may be performed. The results can be reviewed by the analyst with the aim of fine-tuning the algorithms. Offline data analysis is valuable, but it does not address real-time issues. In contrast, online sessions provide a means of BCI system evaluation in a real-world environment. The data are processed in a causal manner and the algorithms are tested in an environment in which the users change over the time as a result of e.g., changes in motivation or fatigue. Although some researchers test new algorithms with only offline data, both offline simulation and online experiments are necessary for effective algorithm design in closed-loop systems. In other words, offline simulation and cross-validation can be valuable methods to develop and test new algorithms, but only online analysis can yield solid evidence of BCI system performance [137,212,213].

Classification algorithms have traditionally been calibrated by users through supervised learning using a labeled data set. It is assumed that the classifier is able to detect the patterns of the brain signal recorded in online sessions with feedback. However, this assumption results in a reduction in the performance of BCI systems, because the brain signals are inherently non-stationary. In this regard, Shenoy *et al.* [214] described two main sources of non-stationarity. On the one hand, the patterns observed in the experimental samples during calibration sessions may be different from those recorded during the online session. On the other hand, progressive mental training of the users or even changes in concentration, attentiveness, or motivation may affect the brain signals. Therefore, adaptive algorithms are essential for improving BCI accuracy. Adaptation to non-stationary signals is particularly necessary in asynchronous and non-invasive BCIs [215,216].

Apart from the fact that supervised learning is not optimal for non-stationary signals classification, large data sets and, thus, long initial calibration sessions are usually required to achieve acceptable accuracy. Semi-supervised learning has been suggested to reduce training time and to update the classifier in the online session on a continuous basis [217]. In semi-supervised learning, the classifier is initially trained using a small labeled data set, after which the classifier is updated with on-line test data.

In a realistic BCI scenario, the signal associated with the subject’s intentions is not usually known and the labels are not available. Either unsupervised learning or reinforcement learning can be applied for BCI adaptation when the labeled data set is not available. Unsupervised methods attempt to find hidden structures in unlabeled data, in order to classify them. Some unsupervised methods rely on techniques for co-adaptive learning of user and machine [218,219] or covariate shift adaptation [220]. Reinforcement learning methods are based on the fact that distinguishing EEG potentials are elicited when a subject is aware of an erroneous decision. These potentials are used as learning signals to prevent that error from being repeated in the future [221].

The adaptation generally results in enhanced performance. Nevertheless, it is worth highlighting that inherent risks exist in an adaptive BCI. A BCI that learns too fast may confuse the user, because training will take place in a changing environment [222]. In addition, adaptive procedures can hide some relevant signal features. Accordingly, there is a tradeoff between highly sensitive adaptation and feature extraction.

Classifiers also have to face two main problems related to the pattern recognition task: the curse of dimensionality and the bias-variance tradeoff. The curse of dimensionality means that the number of training data needed to offer good results increases exponentially with the dimensionality of the feature vector [223]. Unfortunately, the available training sets are usually small in BCI research, because training process takes a long time and is a tiring process for users. The bias-variance tradeoff represents the natural trend of the classifiers towards a high bias with low variance and *vice versa*. Stable classifiers are characterized by high bias with low variance, while unstable classifiers show high variance with low bias. To achieve the lowest classification error, bias and variance should be low simultaneously. A set of stabilization techniques such as the combination of classifiers or regularization can be used to reduce the variance.

The design of the classification step involves the choice of one or several classification algorithms from many alternatives. Several classification algorithms have been proposed such as *k*-nearest neighbor classifiers, linear classifiers, support vector machines, and neural networks, among others. The general trend prefers simple algorithms to complex alternatives. Simple algorithms have an inherent advantage because their adaptation to the features of the brain signal is inherently simpler and more effective than for more complex algorithms. Nevertheless, simple algorithms, whenever outperformed in online and offline evaluations, should be replaced by more complex alternatives [213].

Finally, certain inherent dangers of classification algorithm usage should be pointed out. Although classification algorithms have clearly helped to characterize task relevant brain states, several pitfalls may occur when these algorithms are used by non-experts. Bias and variance of the estimated error of the algorithms, and their overfitting are the main source of difficulties [224]. If a classifier is overfitted, then it will only be able to classify the training data or similar data. Overfitting can be avoided by restricting the complexity of the classification procedure [224]. Classification error is estimated by means of cross validation. Once a classification algorithm is trained, the algorithm is validated on a validation data set, which should be independent of the training data set. This procedure is usually repeated several times, using different partitions of the sample data. The resulting validation errors are averaged across multiple rounds. This approach presents some inherent dangers that must be prevented, because some elements of the partition may not be independent of each other or may not be identically distributed, among other reasons [224]. Next, this section presents the properties of a set of classifiers, in order to make it easier to choose an appropriate classifier for a given type of BCI. All classifier methods are listed in Table 8, along with their main properties.

### 7.1. K-Nearest Neighbor Classifier (k-NNC)

*K*-nearest neighbor classifiers (*k*-NNC) are based on the principle that the features corresponding to the different classes will usually form separate clusters in the feature space, while the close neighbors belong to the same class. This classifier takes *k* metric distances into account between the test samples features and those of the nearest classes, in order to classify a test feature vector. The metric distances are a measure of the similarities between the features of the test vector and the features of each class. The advantage of taking *k* neighbors into account in the classification is that error probability in the decision is decreased. Some training samples may be affected by noise and artifacts, which may seriously influence the classification results. If a decision involving several neighbors is made, then it is less likely that an error will occur, because the probability of several simultaneous erroneous datum is much lower [225].

Rather than only the closest sample, if several *k* closest classes are considered, then a voting scheme is required to decide between competing choices. Since there are no reasons to assume that the distributions of those neighbors are homogenous, it is clear to see that the *k*-NNC has to assign different ranks to the nearest neighbors, according to their distances from the test example. Therefore, *k*-NNC needs to define a weighting function, which varies with the distance in such a way that the output value decreases as the distance between the test feature vector and the neighbor increases. The function defined by Equation (19) [226] meets this requirement:

(19) $$w^{\left(i\right)}=\{\begin{array}{cc} \frac{d^{\left(k\right)}-d^{\left(i\right)}}{d^{\left(k\right)}-d^{\left(1\right)}} & \mathrm{if}\;d^{\left(k\right)}\neq d^{\left(1\right)}\\ 1 & \mathrm{if}\;d^{\left(k\right)}=d^{\left(1\right)} \end{array}$$

where, *d*^(*i*)^ denotes the distance of the *i*-th nearest neighbor from a test example. That is, *d*^(1)^ corresponds to the nearest neighbor and *d*^(*k*)^ to the furthest. The decision rule of *k*-NNC assigns the unknown examples to the class with the greatest sum of weights among its *k* nearest neighbors.

*k*-NNC is not very common in BCI research, because this classifier is very sensitive to the dimensionality of the feature vector [227]. Nevertheless, *k*-NNC has been proven to be efficient with low dimension feature vectors. Also, *k*-NNC has been tested in a multiclass environment [228] and applied to cursor movements on a vertical axis, when classifying SCPs [229].

### 7.2. Linear Discriminant Analysis (LDA)

LDA is a very simple classifier that provides acceptable accuracy without high computation requirements. LDA is very common in the BCI community and is a good choice for designing online BCI systems with a rapid response, but limited computational resources. LDA provides relatively acceptable accuracy and has been used successfully in numerous BCI systems, such as P300 speller [179], multiclass [230], or synchronous [231] BCIs. Nevertheless, it can lead to completely erroneous classifications in the presence of outliers or strong noise [232]. LDA is usually applied to classify patterns into two classes, although it is possible to extend the method to multiples classes [230].

**Figure 5.**
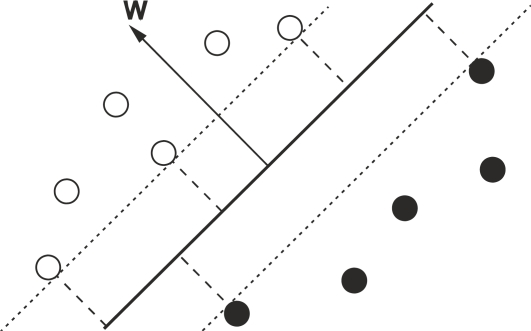
Linear classifier and margins. The decision boundary is the thick line. (adapted from [232]).

For a two-class problem, LDA assumes that the two classes are linearly separable. According to this assumption, LDA defines a linear discrimination function which represents a hyperplane in the feature space in order to distinguish the classes. The class to which the feature vector belongs will depend on the side of the plane where the vector is found (Figure 5). In the case of an *N*-class problem (N > 2), several hyperplanes are used. The decision plane can be represented mathematically as:

(20) $$g(x)=w^{T}x+w_{0}$$

where, *w* is known as the weight vector, ***x*** is the input feature vector and *w_0_* is a threshold. The input feature vector is assigned to one class or the other on the basis of the sign of *g*(***x***).

There are many methods to compute *w*. For example, *w* may be calculated as [233]:

(21) $$w=\Sigma_{C}^{-1}\left(\mu_{2}-\mu_{1}\right)$$

where, *μ_i_* is the estimated mean of class *i* and $\Sigma_{C}=\frac{1}{2}(\Sigma_{1}+\Sigma_{2})$ is the estimated common covariance matrix; the average of the two class empirical covariance matrices. The estimators of the covariance matrix and of the mean are calculated as:

(22) $$\Sigma =\frac{1}{n-1}\sum_{i=1}^{n}\left(x_{i}-\mu \right){(x_{i}-\mu )}^{T}$$

(23) $$\mu =\frac{1}{n}\sum_{i=1}^{n}x_{i}$$

where, ***x*** is a matrix containing *n* feature vectors.*x*_1_, *x*_2_, …, *x_n_* ∈ ℝ*^d^*.

**Figure 6.**
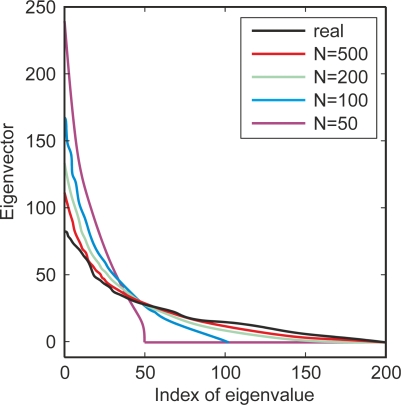
Eigenvalue spectrum of a given covariance matrix (bold line) and eigenvalue spectra of covariance matrices estimated from a finite number of samples (*N* = 50, 100, 200, 500). Note that accuracy increases as the number of trials increase (adapted from [233]).

The estimation of the covariance defined in Equation (22) is unbiased and has good properties under usual conditions. Nevertheless, it may become imprecise in some cases where the dimensionality of the features is too high compared to the number of available trials. The estimated covariance matrix is different from the true covariance matrix, because the large eigenvalues of the original covariance matrix are over estimated and the small eigenvalues are under estimated (Figure 6). It leads to a systematic error which degrades LDA performance [233].

For this reason, a new procedure has been proposed to estimate the covariance, improving the standard estimator defined in the Equation (22). The new standard estimator of the covariance matrix is given by:

(24) Σ(γ)=(1−γ)Σ+γνI

The γ value is referred to as a shrinkage parameter and is tunable between 0 and 1. ν is defined as *trace* (*Σ*)/*d* with *d* being the dimensionality of the features space. The selection of a shrinkage parameter implies a trade-off and is estimated on the basis of the input data [234].

Some improved algorithms have been introduced based on LDA such as Fisher LDA (FLDA) and Bayesian LDA (BLDA) [235]. In the first example, performance was improved by projecting the data to a lower dimensional space, in order to achieve larger intervals between the projected classes and, simultaneously, to reduce the variability of the data in each class. However, FLDA does not work well when the number of features becomes too large in relation to the number of training examples. This is known as the small sample size problem [235].

The second modification can be seen as an extension of FLDA. BLDA solves the small sample size problem by introducing a statistical method known as regularization. The regularization is estimated through Bayesian analysis of training data and is used to prevent overfitting of high dimensional and possibly noisy datasets. Overfitting means the classifier has lost generality and is therefore undesirable in a classifier. If a classifier is overfitted, then it is only able to classify the training data or similar data. In comparison to FLDA, the BLDA algorithm provides higher classification accuracy and bitrates, especially in those cases where the number of features is large [235]. Additionally, BLDA requires only slightly more computation time, which is a crucial requirement in real BCI systems.

### 7.3. Support Vector Machine (SVM)

SVM is a classifier that, in a similar way to LDA classifiers, constructs a hyperplane or set of hyperplanes, in order to separate the feature vectors into several classes. However, in contrast to LDA, SVM selects the hyperplanes that maximize the margins, that is, the distance between the nearest training samples and the hyperplanes [236]. The basis of SVM is to map data into a high dimensional space and find a separating hyperplane with the maximal margin [237] according to Cover’s theorem on the separability of patterns [238]. Cover’s theorem states that a complex classification problem cast in a high-dimensional nonlinear space is more likely to be linearly separable than in a low-dimensional nonlinear space. Also, as for linear analysis classifier, an SVM uses regularization, in order to prevent the classifier from accommodating possibly noisy datasets.

SVM has been used to classify feature vectors for binary [239,240] and multiclass problems [228,230]. It has also been successfully used in a large number of synchronous BCIs [131,230,240]. Such a classifier is regarded as a linear classifier, since it uses one or several hyperplanes. Nevertheless, it is also possible to create a SVM with non-linear decision boundary by means of a kernel function *K(x, y)*. Non-linear SVM leads to a more flexible decision boundary in the data space, which may increase classification accuracy. The kernel that is usually used in the BCI field is the Gaussian or Radial Basis Function (RBF):

(25) $$K(x,y)=\text{exp}\left(\frac{-{\left\|x-y\right\|}^{2}}{2\sigma^{2}}\right)$$

The Gaussian SVM has been applied in BCIs to classify P300 evoked potentials [241–243].

SVM has been widely used in BCI, because it is a simple classifier that performs well and is robust with regard to the curse of dimensionality, which means a large training set is not required for good results, even with very high dimensional feature vectors [228]. These advantages come at the expense of execution speed. Nevertheless, SVM is speedy enough for real-time BCIs [243,244].

### 7.4. Bayesian Statistical Classifier

Bayesian statistical classifier is a classifier which aims to assign, with the highest probability, an observed feature vector ***x*** from its class ***y***. The Bayes’ rule is used to obtain the *a posteriori* probability *P*(*y*|*x*) that a feature vector has of belonging to a given class. Assuming, for example, two classes *L* and *R* corresponding to imaginary left and right movements of the hand, the *a posteriori* probabilities of each class are computed using the Bayes’ rule as:

(26) $$P(y|x)=\frac{P(y)P(x|y)}{P(x)}=\frac{P(y)P(x|y)}{P(x|L)P(L)+P(x|R)P(R)}=\frac{P(x|y)}{P(x|L)+P(x|R)}$$

Typically, it is assumed that the *a priori* probabilities are equal, *P(y) = P(L) = P(R) = 0.5*, since it is supposed the user has no predilection for any movement. In order to calculate the probabilities *P*(*y*|*x*), it is usually supposed that a Gaussian statistical distribution applies to the features for each class, although it may also be assumed that the distribution is a weighted mixture of Gaussian distributions [245]:

(27) $$P(x|y)=\sum_{i=1}^{M}w_{i}P(x|c_{i})$$

where, *w_i_* is the weight of each Gaussian prototype and *M* is the number of prototypes. Two ways are feasible to estimate the Gaussian prototypes mixture [245]. The first is to divide the feature space in several equally sized regions and calculate the mean and variance of the Gaussian prototypes in each area from training data. The set of Gaussian prototypes is equally weighted and the weights *w_i_* are equal to $\frac{1}{M}$. The second uses a Gaussian mixture models (GMM). The different weights *w_i_* and the mean, variance, and covariance matrices that define each Gaussian prototype, are calculated by the expectation maximization (EM) algorithm. EM algorithm is an iterative procedure which guarantees the maximum likelihood or maximum *a posteriori* (MAP) estimates of the parameters in the statistical model. Lui *et al.* [246] made GMM adaptive to significant changes in the statistical distribution of the data during long-term use. In these improvements, the initial mean, variance and covariance of each class is updated over time using a specific number of recent trials.

Bayesian statistical classifiers are not very popular in the BCI community. Nevertheless, they have been used for classifying motor imagery [247] or visual P300 evoked potentials [248].

### 7.5. Artificial Neural Network (ANN)

ANNs are non-linear classifiers that have been used in many applications, in a wide variety of disciplines such as computer science, physics, and neuroscience. The idea of ANNs is inspired in how the brain processes the information. The purpose is to mimic brain activity that immediately solves certain problems, which a conventional computer program processes poorly. For example, ANNs are widely used in pattern recognition, because they are capable of learning from training data. The ability to learn from examples is one of most important properties of ANNs. Once trained, the ANNs are capable of recognizing a set of training data-related patterns. ANNs are therefore associated with BCI applications, since pattern recognition is performed to ascertain user intentions.

An ANN comprises a set of nodes and connections that are modified during the training process. The ANN is fed on a set of training examples and the output is observed. If the output is incorrect, then the internal weights are modified by the training algorithm to minimize the difference between desired and actual output. This training continues until the network reaches a steady state, where no further significant improvement is achieved. In this state, not only should the ANN produce correct outputs for all examples of the training set, but also for inputs that were not encountered during training.

From a mathematical point of view, ANNs define a mapping from an input space to an output space, that can be described as a vector-valued function ***y*** = ***f***(***x***), where both ***x*** and ***y*** may be of any dimensionality. The mapping function ***f*** is a combination of mappings, which are individually performed by single nodes or neurons. Each neuron processes the information non-linearly and the resulting mapping is therefore non-linear. This property is important, especially in those cases where the physical mechanism that generates the input signal is non-lineal.

One of the most well-known ANN structures is the multilayer perceptron (MLP) introduced by Rumelhart and McClelland in 1986. MLPs are very flexible classifiers that can classify any number of classes and adapt to numerous kinds of problems. In the field of BCIs, MLP have been applied to classify two [249], three [200], and five [250] different tasks, and to design synchronous [251] and asynchronous [215] BCIs. Moreover, MLP has been used for preprocessing EEG signals before the feature extraction step rather than the classification step, in order to improve the separability of EEG features [252].

Besides MLP, different types of ANN architecture have been used in the design of BCI systems such as Probabilistic Neural Networks (PNN) [253, 254], Fuzzy ARTMAP Neural Networks [255], Finite Impulse Response Neural Networks (FIRNN) [251] or Probability estimating Guarded Neural Classifiers (PeGNC) [256].

## 8. BCI Applications

BCIs offer their users new communication and control channels without any intervention of peripheral nerves and muscles. Hence, many researchers focus on building BCI applications, in the hope that this technology could be helpful for those with severe motor disabilities. Various BCI applications have very recently been developed thanks to significant advances in the field of EEG-based BCI. EEG signals are used by most BCI applications, because they offer an acceptable signal quality that combines low cost and easy-to-use equipment. Thanks to BCI applications, it is hoped that the quality of life of severely disabled people can be improved. Likewise, the attention given by caregivers will be less intensive, reducing its costs and making the life of relatives less onerous. Moreover, BCI applications potentially represent a powerful tool for revealing hidden information in the user’s brain that cannot be expressed.

The main target populations for BCI applications fall into three classes. The first group includes Complete Locked-In State (CLIS) patients who have lost all motor control, because they may be at a terminal stage of ALS or suffer severe cerebral palsy. The second group comprises Locked-In State (LIS) patients who are almost completely paralyzed, but with residual voluntary movement, such as eye movement, eye blinks, or twitches with the lip. The third group of potential BCI users includes abled bodied people and those with substantial neuromuscular control, particularly speech and/or hand control. BCI have little to offer to the third group, because they can send the same information much more quickly and easily via other interfaces, rather than a BCI. Despite this, BCIs are increasingly used by healthy people in neuromarketing and video games as a tool to reveal affective information of the users, which cannot be so easily reported through conventional interfaces. Likewise, BCI can be used for some people that suffer from neurological disorders such as schizophrenia or depression.

**Figure 7.**
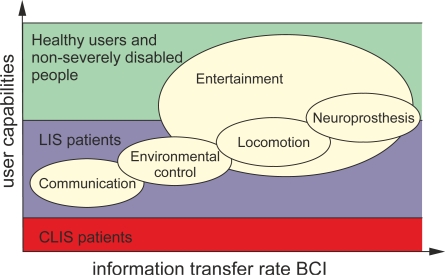
Relationship between BCI application areas, BCI information transfer rates and user capabilities. Horizontal axis: information transfer rate that would make the application controllable. Vertical axis: the degree of capability.

The level of impairment of the potential target population is related to the performance of a BCI system. Kübler *et al.* [257] reported a strong correlation between physical impairment and BCI performance. CLIS patients were unable to control a BCI. Voluntary brain regulation for communication was only possible in LIS patients. However, considering only LIS patients, this relationship between physical impairment and BCI performance disappeared. Figure 7 shows the relationship between BCI application areas and BCI information transfer rates and user capabilities.

It is currently unclear whether BCI technology will ever outperform other established technologies that include eye or muscle-based devices. Currently the latter devices tend to be easier to use and offer better benefit/cost ratios [258,259]. For example, the detection of eye movement is quicker, easier, and more accurate than the detection of ERP modulations. A spelling rate of 10 words per minute can be obtained with unimpaired eye movement, by means of an eyetracker [260]. In that regard, hybrid BCI systems have been proposed to improve performance. They are the combination of two different kinds of BCIs or the combination a BCI with other existing assistive technology [261]. Unless the performance of BCI systems improves considerably, BCI as assistive technology may only be especially attractive for severely disabled people, when other technologies are unsuitable.

At present, LIS patients and those likely to develop CLIS constitute the principal candidates for BCI. Despite the low information transfer rates provided by BCI, the high grade of disability among LIS patients force them to use a BCI rather than more reliable conventional interfaces, such as muscle or eye-gaze based system. Eye-gaze control constraints in some LIS patients are an important issue, because they are obliged to use BCIs that does not depend on eye-gaze control [262,263]. Also, eye-gaze control constraints make some BCI applications more difficult, such as steering a wheelchair.

Nowadays, there are a vast number of very different BCI applications, such as word processors, adapted web browsers, brain control of a wheelchair or neuroprostheses, and games, among others. However, most applications have solely been designed for training or demonstration purposes. Despite the most recent significant advances in BCI technology, there are still many challenges to employing BCI control for real-world tasks [264]: (i) the information transfer rate provided by BCIs is too low for natural interactive conversation, even for experienced subjects and well-tuned BCI systems; (ii) the high error rate further complicates the interaction; (iii) BCI systems cannot be used autonomously by disabled people, because BCI systems require assistants to apply electrodes or signal-receiving devices before the disabled person can communicate; (iv) a BCI user may be able to turn the BCI system off by means of brain activity as input, but usually cannot turn it back on again, which is termed the “Midas touch” problem; and (v) handling BCI applications demands a high cognitive load that can usually be achieved by users in quiet laboratory environment, but not in the real world. Nevertheless, despite all these challenging difficulties, the first steps on the path to long-term independent home use of BCIs have already been taken [12].

Before describing the practical usage of BCI applications, it is worth considering the distinction between BCIs and their applications [8]. As a tool that executes a specific function, particular BCI specifications correspond to the way it performs that function. These specifications can therefore be applied to wide variety of applications, even though the function remains unchanged. The important thing in BCI evaluation is its performance when executing its specific function. In contrast, applications are described in terms of the tools they employ and the purposes they serve. Therefore, BCI evaluation focuses on how well it performs its purpose. In other words, the term BCI refers to the system that records, analyses, and translates the input into commands and the term application denotes the environment in which the BCI estimated output commands are applied. Consequently, the evaluation procedures for BCI systems and their applications differ in each case. The following sub-sections briefly describe BCI applications, classified into five main areas: communication, motor restoration, environmental control, locomotion and entertainment.

### 8.1. Communication

BCI applications for communication deal with severe communication disabilities resulting from neurological diseases. This kind of application probably represents the most pressing research in the field of BCI, because communication activity is essential for humans. Applications for communication purposes outline an operation that typically displays a virtual keyboard on screen, where the user selects a letter from the alphabet by means of a BCI. The distinguishing element in each approach is usually the BCI and the type of control signal.

Voluntary control of SCPs may be used for letter selection. With extensive training, completely paralyzed patients are able to produce positive and negative changes in their SCP to drive the vertical movement of a cursor [117]. Based on this kind of control signal, Birbaumer *et al.* [265] developed a spelling device with an on-screen display, which used a cursor to select letters of the alphabet. Trials involving two patients at advanced stages of ALS showed that they achieved a rate of about 2 characters per minute when writing text messages. Other types of control signals, such as detection of eye blinks [266], which normally represent an artifact in EEG signals, or classification of three mental tasks [215], are also used to select the blocks or characters in a virtual keyboard. Both approaches are nearly the same apart from the control signal. In both cases, the virtual keyboard consisted of a total of 27 symbols, 26 English letters plus the space to separate words, organized in a three row by nine column matrix. Likewise, both applications were based on the same protocol of writing a single letter, which required three steps. Firstly, the whole keyboard was divided into three blocks, each with nine letters each. Then, the user could select a set of nine letters by producing a single, two or three eye blinks [266] or imagining one of three available tasks [215] depending on the case. After the first selection, the set of nine letters was distributed into three subsets, each with three letters, and once again the user again selected one of them. Finally, at the third level, the user chose a single letter amongst the three remaining symbols. The correct spelling rate of each speller was one character per minute using blinks [266] and 2.73 characters per minute for three mental tasks [215].

Obermaier *et al.* [267] also designed a letter spelling based on standard Graz-BCI which also included a virtual keyboard. The letter selection protocol is very similar to the approaches discussed above, except that the entire alphabet consisted of 32 letters and was divided into two halves at each step. In this case, the user chooses either subset of letters by EEG modulation through mental hand and leg motor imagery. The spelling rate achieved by three healthy users varied between 0.5 and 0.85 letters per minute. This is a lower rate than in previous cases, nevertheless, it appears easy to increase the number of letters spelled per minute just by expanding the number of classes to more than two.

**Figure 8.**
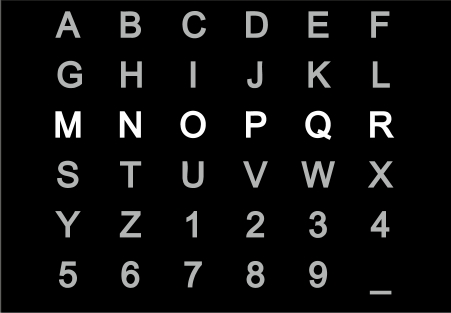
Original P300 speller. Matrix of symbols displayed on a screen computer which serves as the keyboard or prosthetic device (adapted from [123]).

P300 event-related brain potentials are also very popular in BCI letter spelling applications. P300-based BCIs have been proven sufficiently suitable for ALS patients in the early and middle stages of the disease [268]. Besides, this kind of BCI is very handy because the P300 response occurs spontaneously and consequently does not require substantial training. Furthermore, recent progress with P300-based spellers have allowed the development of commercial applications available to the general public [269]. One of the best-known P300 spellers was designed by Farwell and Donchin in 1988 [123]. In this speller, the 26 letters of the alphabet, together with several other symbols and commands, are displayed on-screen in a 6 × 6 matrix (Figure 8) with randomly flashing rows and columns. Then, the user focuses attention on the screen and concentrates successively on the characters to be written, while the EEG response is monitored. Two P300 are elicited for each looked-for element on the matrix, when the desired row or column flashed, thereby allowing the system to identify the desired symbol. The results of the Farwell-Donchin speller trials involving 4 healthy people yielded an acceptable spelling rate of about 2 characters per minute.

**Figure 9.**
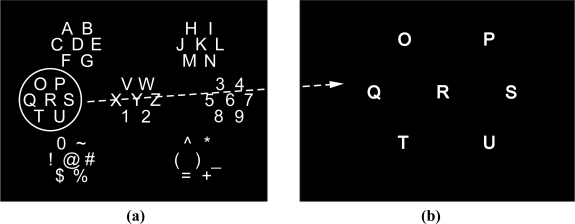
The proposed region-based paradigm for the improved P300 speller: (**a**) The first level of intensification where each group contains up to seven characters; and (**b**) One region is expanded at the second level (adapted from [270]).

The Farwell-Donchin speller provides a relatively high rate and accuracy, but its precision can be improved by reducing perceptual errors in the Farwell-Donchin paradigm [270]. Perceptual error happens when a P300 response is elicited due to flashing rows or columns adjacent to the target symbol, an issue which is its major source of error. Hence, a new letter distribution was presented to overcome this problem (Figure 9) [270]. The idea is to have several regions flashing instead of using rows and columns. The characters are placed into a two-level distribution. At the first level, the characters are distributed into seven groups, each with seven characters, which are also flashing randomly. The group containing the target character is found by P300 detection. At the second level, the characters in the detected group are repositioned and the level one procedure is repeated, and so on until the target character is finally selected.

Townsend *et al.* [137] presented a newly enhanced BCI based on a checkerboard paradigm instead of the standard row/column paradigm introduced by Farwell and Donchin. In this new approach, the standard matrix containing the targets was superimposed on a checkerboard. Trials with advanced ALS patients and healthy people showed a significantly higher mean accuracy for the checkerboard paradigm than for the row/column paradigm. Ahi *et al.* [271] also recently improved the Farwell-Donchin P300 speller by introducing a dictionary to decrease the number of misclassifications in the spelling. The dictionary was used for checking the candidate word proposed by the classifier of P300 responses. In case of misspelling, the dictionary gave a certain number of suggestions from which the system could select. Additionally, in order to reduce the probability of misspelling due to perceptual errors, the usual letter position in the matrix was changed according to the analysis of word similarities in the constructed dictionary.

All previous P300 spellers are based on the recording of visual event-related brain potentials. However, there is no sense in using visual stimuli in cases of severely paralyzed patients with impaired vision or poor control over eye movements. In these cases, auditory stimulation is used in order to make P300 spellers suitable for this group of patients [129,272–274].

Other important applications of communication-related BCIs are Internet browsers adapted to users with severe disabilities because, over the last decade, the Internet has become a very important part of daily life. In this area, “Descartes” is one of the first EEG-controlled web browsers which can be operated by SCPs [275]. Its browser interface is based on arranging the links alphabetically in a dichotomous decision tree, where the user selects or rejects each item, producing positive or negative SCP shifts. “Descartes” presents the shortcoming that only a limited number of web pages can be browsed, because the user receives a number of predefined links arranged in a tree at the start of the web surfing. Besides, graphical links cannot be chosen since the textual label is used to identify the link. A more advanced prototype, called “Nessi”, overcomes these shortcomings thanks to a better user interface [276]. Colored frames are placed around links or selectable items on the web page instead of arranging the links in a tree. More recently, evoked potentials are also used to enhance browser functionality. Mugler *et al.* [128] built an Internet browser with P300 control where the options are all presented as icons in an 8x8 matrix. Jinghai *et al.* [103] developed a browser based on VEPs. One of the advantages of ERPs is that they occur quickly and can lead to relatively high web surfing speeds.

### 8.2. Motor Restoration

Spinal cord injury (SCI) or other neurological diseases with associated loss of sensory and motor functions dramatically decrease the patient’s quality of life and create life-long dependency on home care services. Motor restoration may alleviate their psychological and social suffering. Restoring movement, such as grasping, is feasible in quadriplegic patients through neuroprostheses guided by functional electrical stimulation (FES). FES compensates for the loss of voluntary functions by eliciting artificial muscle contractions. Electrical currents generate artificial action potential by depolarizing intact peripheral motor nerves that innervate the targeted muscle and cause a muscle contraction (see [277] for a review). EEG-based BCI can be used to generate a control signal for the operation of FES, because EEG signals are unaffected by electrical activation of upper extremity muscles [278]. Thanks to their merging of BCI and FES, Pfurtscheller *et al.* [279] developed an application where a tetraplegic patient, suffering from a traumatic spinal cord injury, was able to control paralyzed hands to grasp a cylinder. In that application, the patient generated beta oscillations in the EEG by foot movement imagery. Then, the BCI analyzed and classified the beta burst and the output signal was used to control the FES device that activated the extremity. Also, FES has been used for rehabilitation training after a stroke. Hu *et al.* [280] developed a combined FES-robot system which was continuously driven by the user’s residual electromyography on the affected side for wrist joint training after a stroke, in order to involve the user’s own neuromuscular effort during the training.

FES has been proven to be an effective way to restore movement. Nevertheless, FES requires the use of residual movements, which are not possible in severely injured patients. For this reason, some groups have started to explore approaches that couple neuroprostheses and BCI without FES intervention. Pfurtscheller *et al.* [281] demonstrated that a tetraplegic patient, whose residual upper-limb muscle activity was restricted to the left biceps, due to an upper spinal cord injury, could effectively control a hand orthosis using changes in Rolandic oscillations, which were produced by motor imagery. A lengthy training period was required to use this application. However, the patient was finally able to open and close the hand orthosis almost without any errors. Some years later, the same group validated the coupling of EEG-based BCIs and an implanted neuroprosthesis giving further evidence that BCI is a feasible option for the control of a neuroprostheses [282]. In this study, BCI classified distinctive EEG-patterns that involved power decreases in certain specific frequency bands. These patterns were generated by the user from mental imagery of his paralyzed left hand in motion.

More recently, ERPs are also used to provide motor restoration. Muller *et al.* [13] presented a novel neuroprosthetic device for the restoration of the grasp function for people spinal cord injuries. This neuroprosthetic device consisted of a dual-axis electrical hand prosthesis controlled by BCI based on four-class SSVEPs. Hence, it is possible to select only four movements according to the four LEDs flickering in different frequencies. The user’s gaze shifted between the different LEDs in order to select a movement. One light on the finger index flickering at 6 Hz and another light on the pinky finger flickering at 7 Hz served to turn the hand in supination or pronation. The two remaining lights on the wrist flickering at 8 Hz and 13 Hz represented the orders to open and close each hand.

Within the field of BCI application in motor restoration, BCI systems have been also applied for movement reconstruction in patients with severe post-stroke motor disability. BCI training is hypothesized to provide feedback to sensorimotor cortex and, by doing so, movement is restored as cerebral pathways reorganize to link up motor commands with motor movements. Buch *et al.* [283] developed a BCI system that used MEG activity evoked by patient intent to move a completely paralyzed hand, in order to control grasping motions of a mechanical orthosis attached to the affected hand. Thanks to the hand prosthesis attached to the paralyzed hand and using visual feedback, the patient could learn to open a hand by increasing SMR over the injured hemisphere and to close the hand by decreasing it. MEG provides a much larger and more localized SMR response, which means that even a digit finger may be controlled [284].

MEG-based BCI is too expensive for widespread applications. For that reason, Broetz *et al.* [285] proposed a combination of MEG and EEG-based BCIs. Initially, the MEG-based BCI was used to boost rehabilitation training success. Later, the user continued rehabilitation training with an EEG-based BCI; a more affordable technology than MEG. Finally, the patient practiced physiotherapy training. The results of this study suggest that the combination of BCI training with goal-directed active physical therapy improves the motor abilities of chronic stroke patients. In similar experiments, Caria *et al.* [286] reaffirmed the success of a combination of BCI training and physiotherapy. This study encourages further research on the role of BCIs in brain plasticity and post-stroke recovery.

### 8.3. Environmental Control

One of the main goals of BCI-based applications is to achieve maximum independence for the patient, despite any motor disability. People who suffer severe motor disabilities are often homebound and for this reason, environmental control applications focus on the control of domestic devices such as TV, lights or ambient temperatures. Apart from improving the quality of life of severely disabled people, assistive devices mean that the tasks of the caregiver are less intensive, costs are reduced, and the life of relatives is less onerous.

Cincotti *et al.* [14] presented a pilot study dealing with the integration of BCI technology into the domestic environment. In this study, fourteen patients with severe motor disabilities, due to progressive neurodegenerative disorders, tested a device that provided environmental control through an interface designed to support different levels of motor capacities for each user. Typical peripherals such as keyboard, mouse or joystick were offered to allow the device control through upper limb residual motor abilities. Head trackers and microphones for voice recognition were also available in cases of people with impaired limbs but intact neck muscles and comprehensive speech. Lastly, in cases of totally disabled people, the system could be controlled by voluntary modulations of sensorimotor rhythms recorded by the EEG-based BCI. Thereby, the application offered the patient different access modalities that matched their gradual loss of motor abilities due to progressive neurodegenerative diseases. As output devices, the system allowed the use of a basic group of domestic appliances such as lights, TV and stereo sets, a motorized bed, an acoustic alarm, a front door opener, and a telephone, as well as wireless cameras to monitor the surrounding environment.

Invasive techniques have also been proposed in environmental control applications. Hochberg *et al.* [287] implanted BrainGate sensors in the primary motor cortex of a tetraplegic patient to control a cursor. The initial trials yielded promising results, where the patient could handle e-mail applications or operate devices such as a television by imagining limb motions, even while conversing.

### 8.4. Locomotion

BCI applications that allow disabled people to control a means of transportation represent an important field in their use. Thanks to these applications, people suffering from paraplegia or with other physical impairments can autonomously drive a wheelchair, making them more autonomous and improving their life quality. Portability is a necessity for these kinds of applications. Hence the use of a BCI based on EEG recording is enforced. EEG signals are typically very noisy and are highly variable, which means a relatively long time between commands that will be of high uncertainty. Therefore, the main challenge is to achieve sufficient accuracy in driving as well as reaching real-time control, in spite of the ultra-low information transfer rates provided by BCI. For this reason, some studies on BCIs proposed invasive techniques to record EEG signals, because they achieved more spatial resolution and reduced noise. Serruya *et al.* [288] experimented with monkeys implanting an electrode array in the motor cortex. These initial experiments showed that the monkeys were able to move a computer cursor to any position, opening up new human applications.

However, the risks related to invasive BCIs lead research towards building non-invasive applications for human use. Some pilot experiments concerned with locomotion illustrate the feasibility of using EEG signals for continuous control of a mobile robot in an indoor environment with several rooms, corridors, and doorways [215,289]. The results of these experiments opened the possibility for physically disabled people to use a portable EEG-based BCI for controlling wheelchairs. To the best of our knowledge, in 2005, Tanaka *et al.* [127] presented the first application of wheelchair control using only EEG. In this study, the surrounding floor was divided into squares between which the user decided to move by imagining left or right-limb movements. Driven by user decisions, the wheelchair therefore moved from one square to another. Tests with six healthy subjects were quite encouraging and demonstrated the viability of wheelchairs control solely through the use of EEG signals.

In wheelchair control by BCI-based systems, the usual problems are the infrequent control signal and the low information transfer rate and accuracy provided by a BCI. In that respect, some improvements have been presented over the past few years. Synchronous P300-based BCIs have been introduced in order to assure better accuracy. Likewise, to overcome the usual low bit rate in BCIs, the systems have been endowed with certain autonomy, decreasing the number of interactions required. Rebsamen *et al.* [290] designed a simplified wheelchair control by constraining the movements to guidepaths defined by the patient or a helper. These guidepaths were attached to a specific point in the environment and stored by the system. The user selected the destination through a P300-based BCI and the wheelchair automatically followed the path. The user only had to decide when the wheelchair would stop. For path guidance, the system steering the wheelchair had to be kept informed of its localization uninterruptedly. To that effect, the wheelchair relied on an odometer and a bar-code scanner to read bar-code patterns previously placed on the floor along the paths. Some years later, the system was improved to ensure safer control. Two faster BCIs based on P300 and the µ/β rhythm were employed, allowing the user to stop the wheelchair more quickly [291]. Both applications were tested with healthy people.

The main disadvantage found in the preceding approaches is that the control assistance has little flexibility and is not capable of dealing with unknown and populated scenarios. Iturrate *et al.* [292] overcame this shortcoming by making the system create a dynamic reconstruction of the surrounding scenario. Other studies suggested that help should only be available in those cases where the user experienced more difficulties driving the wheelchair e.g., in a narrow corridor [216,293,294]. Three levels of assistance may be possible in the shared control: collision avoidance, obstacle avoidance and orientation recovery, which are only activated as required by the user [293]. Before executing the user’s steering commands, the share control evaluates the situation from the data provided by a set of laser scanners. Scanners inspect the environment and detect potential obstacles or walls.

### 8.5. Entertainment

Entertainment-orientated BCI applications have typically had a lower priority in this field. Until now, research into BCI technology has usually focused on assistive applications, such as spelling devices, wheelchair control or neuroprostheses rather than applications with entertainment purposes. However, interest in entertainment applications has arisen over the recent years due to the significant advances in this technology. In fact, improvements in its performance have opened the way to extending BCI use to non-disabled people. BCIs create a new interaction modality which may turn video games into even more challenging and attractive experiences. Additionally, BCI may provide a way of accessing knowledge on the user’s experiences, thereby improving games through information from brain activity. BCIs can report when the gamer is bored, anxious or frustrated with the aim of using this knowledge for designing future games [295].

**Figure 10.**
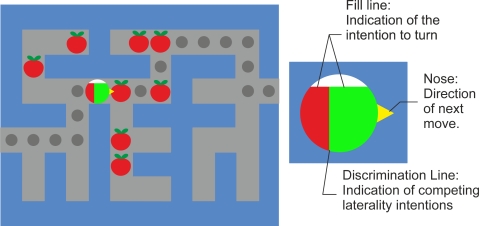
Pacman game. The gamer has to move through the maze to reach the exit in the right wall. The shortest path is marked with gray track marks, but the gamer can decide to run the rest of maze to receive additional credits (adapted from [296]).

Entertainment-oriented BCI applications have adapted very well-known video games such as Pacman, Pong and similar games so that they may be played through motor imagery [296]. By way of illustration, Figure 10 shows a screenshot of a Pacman game. The Pacman makes one step each 1.5–2 s with the aim of giving the gamer enough time to perform a control command. The Pacman’s head is filled with red and green color from bottom up as the player’s intention to turn rises and the yellow nose points to the direction in which the user intends to take the Pacman. In another study, a pinball game was developed, in order to illustrate that it is possible to successfully apply non-invasive recording techniques for complex control tasks [297].

External evoked potentials have been also used for game implementations. Middendorf *et al.* [298] designed a simple flight simulator controlled by a BCI based on Steady State Visual-Evoked Response (SSVER). This simulator was very modest and only two control actions were possible. The position could be moved to the left or the right only. Two methods were tested over the airplane control trials. On the one hand, the control command (right or left) was detected, according to the strength of the SSVERs. And on the other hand, the selection was identified taking into account the frequency of SSVER. The results of the trials with able-bodied participants showed that the last one was preferred because it required little or no training since the system capitalized on the natural occurring responses. Lalor *et al.* [299] presented the “MindBalance” game where six healthy users were asked to keep a tight-rope walker in balance. The application was also based on SSVEP generated in response to phase-reversing checkerboard patterns. By means of other kinds of ERPs, Finke *et al.* [300] implemented the “MindGame” based on P300 events. It was suggested that this game would be a useful tool for attention training, because the P300 potentials are also attention markers.

**Figure 11.**
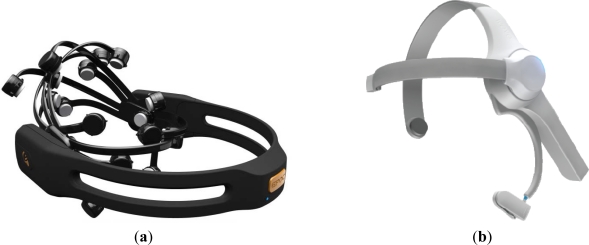
(**a**) Emotiv EPOC neuroheadset [5]; (**b**) Neurosky Mindwave [6].

All previous examples involved experimental games that have only been found useful in a research context. However, there are some companies preparing commercial BCI games for future markets. Emotiv [5] has already developed a numerous set of BCI-based games, such as Cortex Arcade and Spirit Mountain Demo Game, among others. Furthermore, the company sells a low cost BCI with 14 electrodes, the so-called EPOC neuroheadset (Figure 11(a)) which can be bought accompanied by an Application Programming Interface (API). Thanks to this API, the development of the BCI-based applications is made much simpler. The company Neurosky [6] also markets the Mindwave neuroheadset (Figure 11(b)) with software applications that can respond to user brainwaves or mental states. Likewise, it provides a set of software tools for developers. Also, large software companies such as Microsoft have shown interest in BCI research, exploring the development of pilot novel applications that use BCIs [301].

### 8.6. Other BCI Applications

BCI systems have also been used in a broad variety of applications beyond the traditional areas of communication, motor restoration, environmental control, locomotion, and entertainment. The ability of BCI feedback to induce cortical plasticity may be the basis for medical applications. Users can acquire selective control over certain brain areas by means of neurofeedback, with the aim of inducing behavioral changes in the brain. Neurofeedback provided by a BCI system may improve cognitive performance [302,303], speech skills [304], affection [305], and pain management [306], and has been used in the treatment of mental disorders, such as epilepsy [307,308], attention deficit [309], schizophrenia [310], depression [311], alcohol dependence [312], or paedophilia [313]. On the other hand, brain signal recordings can be used in an assessment of brain functions to evaluate their status in health and disease [314].

The opportunity to examine brain signals can also be commercially exploited. Neuromarketing is a relatively young field of research that applies neuroscientific methods to marketing research. To date, few neuromarketing studies have been conducted, although some evidence has been found to suggest that neuroimaging could have a role in several areas of marketing [315–318]. Neuromarketing may provide a more efficient trade-off between costs and benefits. Product concepts could be tested by means of neuromarketing, removing those that are not promising at the start of the manufacturing process. This would lead to a more efficient distribution of sources, because only the more promising products would be developed [319]. In addition, neuromarketing may be a source of more accurate information on the underlying preferences of the users, rather than data from standard market research studies [319]. Neuroimaging may reveal hidden information on consumers’ true preferences that cannot be explicitly expressed. The brain’s response to advertisements could be measured and the effectiveness of advertising campaigns could therefore be quantified.

Despite it being an emerging field, several companies such as Neurofocus [320], Neuroconsult [321], Neuro Insight [322] or EmSense [323], among others, currently offer neuromarketing services. It is also attracting increasing attention among researchers. The field has raised some ethical issues concerning this technology, in as much as it may be able to manipulate the brain and consumer behavior [324].

## 9. Conclusions

This article has reviewed the state-of-the-art of BCI systems, discussing fundamental aspects of BCI system design. The most significant goals that have driven BCI research over the last 20 years have been presented. It has been noted that many breakthroughs were achieved in BCI research. Different neuroimaging approaches have been successfully applied in BCI: (i) EEG, which provides acceptable quality signals with high portability and is by far the most usual modality in BCI; (ii) fMRI and MEG, which are proven and effective methods for localizing active regions inside the brain; (iii) NIRS, which is a very promising neuroimaging method in BCI; and (iv) invasive modalities, which have been presented as valuable methods to provide the high quality signals required in some multidimensional control applications e.g., neuroprostheses control.

A wide variety of signal features and classification algorithms have been tested in the BCI design. Although BCI research is relatively young, many advances have been achieved in a little over two decades, because many of these methods are based on previous signal processing and pattern recognition research. Many studies have demonstrated the valuable accuracy of BCIs and provided acceptable information bit rate, despite the inherent major difficulties in brain signal processing. Accordingly, user training time has been significantly reduced, which has led to more widespread BCI applications in the daily life of disabled people, such as word processing, browsers, email, wheelchair control, simple environmental control or neuroprostheses among others.

In spite of the recent important advances in the BCI field, some issues still need to be solved. First, the relative advantages and disadvantages of the different signal acquisition methods are still unclear. Their clarification will require further human and animal studies. Second, invasive methods need further investigation to deal with tissue damage, risk of infection, and long-term stability concerns. Electrodes that contain neurotropic mediums that promote neuronal growth and wireless transmission of neuronal signals recorded have already been proposed. Third, the electrophysiological and metabolic signals that are best able to encode user intent should be better identified and characterized. The majority of BCI studies have treated time, frequency, and spatial dimensions of brain signals independently. These signal dimension interdependencies may lead to significant improvement in BCI performance. Fourth, information bit rate provided by current BCIs is low for effective human-machine interaction in some applications. Exogenous-based BCI may provide much higher throughput. Fifth, the unsupervised adaptation is a key challenge for BCI deployment outside the lab. Some moderately successful adaptive classification algorithms have already been proposed. And finally, most BCI applications are at the research stage and they are not ready to be introduced into people’s homes for continuous use in their daily life. In addition to their low information transfer rates and variable reliability, most current BCI systems are uncomfortable, because the electrodes need to be moistened, the software may require initiation, and the electrode contacts need continuous correction. An easy-to-use P300-based BCI with remote monitoring using a high-speed internet connection has already been proposed to reduce dependence on technical experts.

The latest advances in BCI research suggest that innovative developments may be forthcoming in the near future. These achievements and the potential for new BCI applications have obviously given a significant boost to BCI research involving multidisciplinary scientists e.g., neuroscientists, engineers, mathematicians, and clinical rehabilitation specialists, among others. Interest in the BCI field is expected to increase and BCI design and development will in all probability continue to bring benefits to the daily lives of disabled people. Furthermore, recent commercial interest within certain companies suggests that BCI systems may find useful applications in the general population, and not just for people living with severe disabilities. In the near future, BCI systems may therefore become a new mode of human-machine interaction with levels of everyday use that are similar to other current interfaces.

## Figures and Tables

**Table 1.** Summary of neuroimaging methods.

**Neuroimaging method**	**Activity measured**	**Direct/Indirect Measurement**	**Temporal resolution**	**Spatial resolution**	**Risk**	**Portability**

**EEG**	Electrical	Direct	∼0.05 s	∼10 mm	Non-invasive	Portable
**MEG**	Magnetic	Direct	∼0.05 s	∼5 mm	Non-invasive	Non-portable
**ECoG**	Electrical	Direct	∼0.003 s	∼1 mm	Invasive	Portable
**Intracortical neuron recording**	Electrical	Direct	∼0.003 s	∼0.5 mm (LFP)	Invasive	Portable
				∼0.1 mm (MUA)		
				∼0.05 mm (SUA)		
**fMRI**	Metabolic	Indirect	∼1 s	∼1 mm	Non-invasive	Non-portable
**NIRS**	Metabolic	Indirect	∼1 s	∼5 mm	Non-invasive	Portable

**Table 2.** Summary of control signals.

**Signal**	**Physiological phenomena**	**Number of choices**	**Training**	**Information transfer rate**

VEP	Brain signal modulations in the visual cortex	High	No	60–100 bits/min
SCP	Slow voltages shift in the brain signals	Low (2 or 4, very difficult)	Yes	5–12 bits/min
P300	Positive peaks due to infrequent stimulus	High	No	20–25 bits/min
Sensorimotor rhythms	Modulations in sensorimotor rhythms synchronized to motor activities	Low (2, 3, 4, 5)	Yes	3–35 bits/min

**Table 3.** Features of VEP modulations: t-VEP, f-VEP and, c-VEP.

**VEP modulation**	**Features**
t-VEP	– Relatively low information transfer rate (<30 bits/min) – Synchronous signal is necessary – No user training required
f-VEP	– High information transfer rate (30–60 bits/min) – Simple system configuration – No user training required – More suitable for application with few options
c-VEP	– Very high information transfer rate (>100 bits/min) – Synchronous signal is necessary – User training required – More suitable for application with many options

**Table 4.** Main differences between exogenous and endogenous BCI.

**Approach**	**Brain signals**	**Advantages**	**Disadvantages**

Exogenous BCI	– SSVEP – P300	– Minimal training – Control signal set-up easily and quickly – High bit rate (60 bits/min) – Only one EEG channel required	– Permanent attention to external stimuli – May cause tiredness in some users
Endogenous BCI	– SCPs – Sensorimotor rhythms	– Independent of any stimulation – Can be operated at free will – Useful for users with sensory organs affected – Suitable for cursor control applications	– Very time-consuming training (months or weeks) – Not all users are able to obtain control – Multichannel EEG recordings required for good performance – Lower bit rate (20–30 bits/min)

**Table 5.** Main differences between synchronous and asynchronous BCIs.

**Approach**	**Advantages**	**Disadvantages**

Synchronous BCI	– Simpler design and performance evaluation – The user can avoid generating artifacts since they can perform blinks and other eye movements when brain signals are not analyzed	– Does not offer a more natural mode of interaction
Asynchronous BCI	– No requirement to wait for external cues – Offers a more natural mode of interaction	– Much more complicate design – More difficult evaluation

**Table 6.** Summary of feature extraction methods.

	**Method**	**Properties**	**Applications**
**Dimension reduction**	PCA	– Linear transformation – Set of possibly correlated observations is transformed into a set of uncorrelated variables – Optimal representation of data in terms of minimal mean-square-error – No guarantees always a good classification – Valuable noise and dimension reduction method. PCA requires that artifacts are uncorrelated with the EEG signal	[155,157,158]
	ICA	– Splits a set of mixed signals into its sources – Mutual statistical independence of underlying sources is assumed – Powerful and robust tool for artifact removal. Artifacts are required to be independent from the EEG signal – May corrupt the power spectrum	[160,161,164–168]
**Space**	CSP	– Spatial filter designed for 2-class problems. Multiclass extensions exist – Good result for synchronous BCIs. Less effective for asynchronous BCIs – Its performance is affected by the spatial resolution. Some electrode locations offer more discriminative information for some specific brain activities than others – Improved versions of CSP: WCSP, CSSP, CSSSP	[183–187]
**Time-frequency**	AR	– Spectrum model – High frequency resolution for short time segments – Not suitable for non-stationary signals – Adaptive version of AR: MVAAR	[170,172]
	MF	– Detects a specific pattern on the basis of its matches with predetermined known signals or templates – Suitable for detection of waveforms with consistent temporal characteristics	[151,173]
	CWT	– Provides both frequency and temporal information – Suitable for non-stationary signals	[179,180]
	DWT	– Provides both frequency and temporal information – Suitable for non-stationary signals – Reduces the redundancy and complexity of CWT	[181,182]

**Table 7.** Summary of feature extraction methods.

	**Method**	**Properties**	**Applications**
**Features selection**	GA	– High resource consumption – Possible premature convergence	[188,189]
	SFS/SBS	– Suboptimal methods	[191,192]
	SFFS/SBFS	– Modified versions of SFS/SBS methods – Based on *plus l-take away r* algorithm – Partially overcome the deficiencies of SFS/SBS	[194–196]

**Table 8.** Summary of classification methods.

	**Approach**	**Properties**	**Applications**
**Generative model**	Bayesian analysis	– Assigns the observed feature vector to the labeled class to which it has the highest probability of belonging – Produces nonlinear decision boundaries – Not very popular in the BCI systems	[245–248]
**Linear**	LDA	– Simple classifier with acceptable accuracy – Low computation requirements – Fails in the presence of outliers or strong noise. Regularization required – Usually two class. Extended multiclass version exits. – Improved LDA versions: BLDA, FLDA	[179,230,231,233–235]
	SVM	– Linear and non-linear (Gaussian) modalities – Binary or multiclass method – Maximizes the distance between the nearest training samples and the hyperplanes – Fails in the presence of outliers or strong noise. Regularization required – Speedy classifier	[131,228,230, 237,239–244]
**Non-linear**			
	k-NNC	– Uses metric distances between the test feature and their neighbors – Multiclass – Efficient with low dimensional feature vectors. Very sensitive to the dimensionality of the feature vectors	[227–229]
	ANN	– Very flexible classifier – Multiclass – Multiple architectures (PNN, Fuzzy ARTMAP ANN, FIRNN, PeGNC)	[200,215, 249–256]
